# The preferred nucleotide contexts of the AID/APOBEC cytidine deaminases have differential effects when mutating retrotransposon and virus sequences compared to host genes

**DOI:** 10.1371/journal.pcbi.1005471

**Published:** 2017-03-31

**Authors:** Jeffrey Chen, Thomas MacCarthy

**Affiliations:** Department of Applied Mathematics and Statistics, Stony Brook University, Stony Book, New York, United States of America; Fred Hutchinson Cancer Research Center, UNITED STATES

## Abstract

The AID / APOBEC genes are a family of cytidine deaminases that have evolved in vertebrates, and particularly mammals, to mutate RNA and DNA at distinct preferred nucleotide contexts (or “hotspots”) on foreign genomes such as viruses and retrotransposons. These enzymes play a pivotal role in intrinsic immunity defense mechanisms, often deleteriously mutating invading retroviruses or retrotransposons and, in the case of AID, changing antibody sequences to drive affinity maturation. We investigate the strength of various hotspots on their known biological targets by evaluating the potential impact of mutations on the DNA coding sequences of these targets, and compare these results to hypothetical hotspots that did not evolve. We find that the existing AID / APOBEC hotspots have a large impact on retrotransposons and non-mammalian viruses while having a much smaller effect on vital mammalian genes, suggesting co-evolution with AID / APOBECs may have had an impact on the genomes of the viruses we analyzed. We determine that GC content appears to be a significant, but not sole, factor in resistance to deaminase activity. We discuss possible mechanisms AID and APOBEC viral targets have adopted to escape the impacts of deamination activity, including changing the GC content of the genome.

## Author summary

The APOBEC family of cytidine deaminases are important enzymes in most vertebrates. The ancestral member of this gene family is *activation induced deaminase* (AID), which mutates the Immunoglobulin loci in B Cells during antibody affinity maturation in jawed vertebrates. The APOBEC family has expanded particularly in the mammals and in primates, where they have evolved roles in restriction of viruses and retrotransposons. Biochemical studies have established that AID preferentially targets “hotspots” such as AGC and avoids “coldspots” such as CCC. Other APOBECs have evolved distinct hotspots. For example, APOBEC3G, which targets retroviruses including HIV, has evolved to target the motif CCC as a hotspot, but it is unclear why. Here we ask why the AID/APOBEC cytidine deaminases evolved their particular mutational hotspots. Our results show that a wide range of unrelated genes including mammalian LINE1 ORFs and non- mammalian (ancestral-like) viruses are highly susceptible to mutations in APOBEC hotspots and less susceptible to the hypothetical non-APOBEC hotspots. On the other hand, mammalian viruses tend to exhibit low susceptibility to the same APOBEC hotspots, suggesting these viruses have co-evolved to minimize potential damaging mutations, and that the native GC content plays a large role in this behavior.

## Introduction

The AID/APOBEC family of cytidine deaminases have important functions in both intrinsic and adaptive immunity. AID is expressed primarily in germinal center B cells as part of the adaptive immune response [1], whereas the APOBECs act primarily in the intrinsic immune response in various cell types (reviewed in [2]). These mutagenic enzymes act mostly upon single-stranded DNA [3] converting Cytosine in DNA or RNA to Uracil, or for AID, a methylated Cytosine to Thymine *in vitro* [4]. In the absence of further editing, the resulting U-G mismatch in DNA will often be replicated over to create a C to T transition mutation [5]. AID in particular relies on downstream non-canonical DNA repair pathways to introduce further mutations [6]. Although AID-mediated mutations occur at rates approximately 10^6^ higher than background [7, 8], they occur almost entirely in the Immunoglobulin genes that code for the B-cell receptor [9, 10]. At the same time, DNA editing mechanisms can potentially target the host genome (reviewed in [11]) and evidence of AID and APOBEC-mediated mutagenesis has been identified in many human cancers [12]. Thus, there appears to be a tradeoff between the benefits and the potential for self-damage of APOBEC-mediated mutations, yet the details on how this tradeoff is achieved both on a biochemical and evolutionary level are still not well understood.

AID has been proposed to be the most ancestral member of the APOBEC family [13] and is found in all jawed vertebrates. Duplication and diversification of AID have created the APOBEC family, which edit both RNA and DNA. All mammals additionally have at least 4 APOBEC genes, APOBECs1-4 [14, 15]. APOBEC1 deaminates the mRNA of the Apolipoprotein B gene (ApoB) at a specific site to introduce a stop codon in humans and mice [16, 17]. Zebrafish APOBEC2 appears to maintain embryonic development and other functions in development including retina regeneration [18–20], while the role of human APOBEC2 is currently unknown. The function of the APOBEC4 gene also still not known [2].

APOBEC3 has diversified considerably during mammalian evolution. Whereas mice have a single APOBEC3, humans have seven APOBEC3 variants (A-H) [21]. The APOBEC3 genes participate widely in innate immunity by mutating retrotransposons [22], exogenous viruses [23], and endogenous viruses [24].

Human APOBEC3s differ in their restriction capabilities. Perhaps the best understood example of APOBEC restriction is against HIV, which is targeted by human APOBEC3G. The HIV genome in turn encodes a counter-defense in the form of the *vif* gene, which encodes a protein that targets APOBEC3G for degradation. Experiments *in vitro* using *vif*-deficient viruses showed that HIV was highly restricted and mutated by APOBEC3G [25], and later APOBEC3F [26]. Although APOBEC deamination appears effective in inhibiting HIV in the absence of *vif*, other studies suggest that HIV has evolved its own deamination hotspots, thus co-opting APOBEC mutagenesis to increase the likelihood of beneficial mutations [27]. Thus, there is a conflict between HIV adopting APOBEC3G mutation via hotspots compared to the ability of APOBEC to induce lethal hypermutation, although it is not currently established to what extent HIV favors either. Anti-viral activity for human APOBEC3G and other mammalian APOBEC3s has been observed across a wide range of viruses including Human Papillomaviruses such as HPV-16 [28], Adeno-associated virus (AAV) [29], Torque Teno Virus (TTV) [30], Human Herpes-Simplex Virus 1 (HSV-1)[31], Human T-lymphotropic virus 1 (HTLV-1) [32] and Simian Foamy Virus (SFV) [33]. AID has been shown to deaminate the Hepatitis B Virus (HBV) [34] and C virus (HCV) *in vitro* [35]. In addition, APOBECs are thought to restrict human endogenous retroviruses (HERV) [24]. Furthermore, human APOBEC3s are capable of mutating the Long Terminal Repeat (LTR) retrotransposons such as the LINE1 (L1) element [36]. In particular, human APOBEC3B, human APOBEC3C, and human APOBEC3F strongly inhibit L1 retrotransposition *in vitro*, while human APOBEC3G and human APOBEC3H weakly inhibit this same activity [37]. This phenomenon has also been demonstrated with APOBEC3s in reptiles [38]. Homologs of human APOBEC3B have been shown to restrict L1 retrotransposition in other primates as well, where greater L1 activity was correlated with lower human APOBEC3B expression levels and diversity [39].

AID and the human APOBEC3s differ not only by their targets which include exogenous and endogenous viruses, but also their preferred deamination context in DNA or RNA. The relation between these preferred contexts and their biological targets is still poorly understood. Although members of the APOBEC family all mutate C to U, individual preferences vary. AID, for example, preferentially deaminates the hotspot WRC (W = A/T, R = A/G, C is mutated) while avoiding the coldspot SYC (S = G/C, Y = C/T). APOBEC3G, on the other hand, preferentially mutates CCC, an AID coldspot [6, 40]. Additionally, in the context of mutations targeting HERV, it has been shown that the +1 position is also important for APOBEC3F and APOBEC3G leading to more complex motifs, respectively TTCA and CCCA [41]. Preferences for AID/APOBEC targeting are summarized in Table 1. Interestingly, individual APOBEC preferences can be changed via minimal amino acid changes in a hotspot recognition loop [42].

**Table 1:** Biochemical properties of different mammalian cytidine deaminases.

Mutagen	Hotspot	Mutation	Expressed in	Targets
AID	WRC [43]	WRT (before further editing)	B-Cells, Breast Tissue[26]	Immunoglobulin [1]
Human APOBEC3B	TC [44]	TT	Some T cells and keratinocytes [26]	Retrotransposons [37]
Human APOBEC3G	CCC [42, 45]	CCT	T cells [46]	HIV [45], HCV [35], HBV [47]
Human APOBEC3F	TTC [42]	TTT	T cells [46]	HIV [48]
Human APOBEC3C	TTC	TTT		HPV [28]
Human APOBEC3A	TC [49]	TT	Keratinocytes [2]	AAV [50], LINE-1 [37], HPV [28]
Mouse APOBEC3	TYC [51, 52]	TYT		

The AID/APOBEC enzymes must maintain a delicate balance of ensuring deamination is sufficient for key functions such as antibody diversification and restriction of viral and retrotransposon activity, while avoiding detrimental targeting of the host genome. Achieving this balance is accomplished via several mechanisms. Firstly, individual AID/APOBECs are expressed in distinct cell types. For example, AID is expressed primarily in Germinal Center B cells, whereas APOBEC3G is expressed in several cell types, particularly T cells [53]. Additionally, there may be intracellular regulation. Thus, in Germinal Center B-cells, AID is mainly found in the cytoplasm but its activity levels, are modulated in part by its Nuclear Localization Signal (NLS) [54]. Although APOBEC3B also contains an NLS domain, this type of regulation has still not been investigated for other APOBECs [55]. Although AID is actively targeted to the antibody (Immunoglobulin) loci in B-cells, it also has the potential to mutate many “off-target” locations throughout the entire genome [56]. These targets include known oncogenes such as Bcl6, Myc, and others. Mistargeted AID is also linked to poor prognosis in Chronic lymphocytic leukemia (CLL) [57] and other B Cell Lymphomas [58]. Furthermore, the mutation pattern of human APOBEC3B, NTC, has been strongly correlated to so-called “kataegis” mutations in breast cancer, characterized by short inter-mutation distances [12].

Although the mechanism of action and physical structure of several APOBECs are reasonably well characterized, it is not well understood how their specific hotspots were established during APOBEC evolution. We investigate the hypothesis that the APOBECs evolved their mutational preferences to control the impact of deaminase activity by evolving preferences that induce the most damage to their intended targets such as exogenous virus genomes, while causing the least damage to the host genes. We describe a bioinformatics-based approach examining the impacts of motifs, both known and hypothetical, on various genomes. We demonstrate that the current APOBEC preferences are shaped by their ability to restrict their targets, though these observed vulnerabilities may be mediated in large part by the GC content of the native sequence.

## Results

### Analysis model

**Fig 1:**
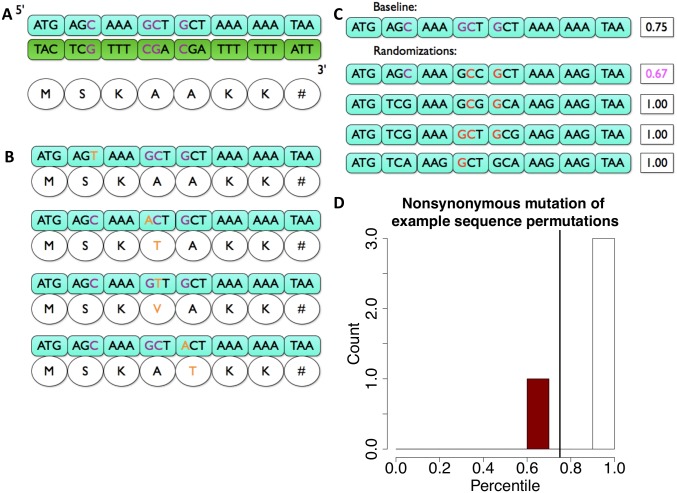
Description of the model used to calculate susceptibility of a sequence to a particular motif. A) The example sequence has 4 AGC motifs, indicated in purple (with two motifs occurring in the reverse orientation as GCT). B) If every AGC and GCT mutation were mutated to AGT and ACT (as indicated in orange) consistent with AID deamination, three out of four of these motifs cause nonsynonymous mutations changing the corresponding amino acid sequence. C) Susceptibility to the AGC motif is calculated by comparing the percentage of random sequences (maintaining protein sequence) with a lower nonsynonymous mutation fraction. D) Since only one random sequence out of four has a lower nonsynonymous mutation fraction than the baseline, this sequence’s percentile, or susceptibility is 0.25.

Our motivation is to quantify the impact of deamination at motifs, both known and hypothetical, across many different potential targets. Previous structural descriptions of APOBEC mutagenesis have indicated the role of a recognition pocket in the -2 and -1 positions on the single-stranded DNA substrate preceding the targeted Cytosine [59]. We therefore examined the effects of mutation at each of the 16 different NNC hypothetical hotspots, referred throughout as NNC motifs (N = any nucleotide) to NNT, consistent with the AID/APOBEC deamination mechanism in the absence of further repair. We measure a genome’s susceptibility to mutation by extending a method first introduced by Karlin [60] (see: “Calculating susceptibility” section in Methods for a detailed definition, including discussion of necessary controls). Briefly, the model we use assesses the impact of a particular hotspot or motif (for example, AGC) on a given gene coding sequence by first counting the number of observed motifs in both orientations, then counting the number of nonsynonymous (changing the amino acid) mutations on the sequence that would occur by mutating C to T in every observed motif (or G to A in the reverse orientation) (Fig 1A). In quantifying a coding sequence’s susceptibility to mutations in a particular motif, we assumed that nonsynonymous mutations have more potential to be deleterious. Given the complexity of nucleotide sequence evolution, there are many factors this simple model does not account for, such as the GC content of the genome, or the impact of hypothetical mutations other than from C to T. To address these considerations, we also evaluate several alternative models (see: “Alternative models and controls corroborate observed susceptibilities” section below).

To illustrate our method, we show an example in Fig 1. Here, the target sequence has 4 AGC motifs, with two on the forward strand, and two on the reverse strand (appearing as the reverse complement of AGC, GCT) of which 3 cause nonsynonymous mutations and 1 is synonymous when mutated from C/G to T/A. This sequence therefore has a nonsynonymous mutation fraction of 0.75, equivalent to the probability that a mutation at an AGC site causes an amino acid change (Fig 1B).

This fraction was then compared to a null model consisting of 1000 randomized sequences that preserve the amino acid sequence, but where synonymous codons are chosen randomly. For each randomized sequence in the null model, the nonsynonymous mutation fraction was calculated. Under the model, if a gene is susceptible to a particular NNC motif, then it would have a higher incidence of nonsynonymous mutations than the majority of randomized versions from the null model. We therefore refer to the percentile of randomized sequences having a lower nonsynonymous mutation fraction than the wild-type sequence as that gene’s susceptibility for a given motif (Fig 1C). It is in fact equivalent to a P-value for susceptibility. Thus, a particular motif is susceptible to mutation if the percentile is close to 1. Conversely, a gene with a low susceptibility, closer to 0, can be considered resistant to that same motif. As an example, if we are considering AGC to AGT mutations, if a gene has a nonsynonymous mutation fraction of 0.75 but in the null model, 1 out of 4 permutations have an even lower nonsynonymous mutation fraction, this gene’s sequence would be at the 25^th^ percentile for the nonsynonymous mutation fraction of the AGC motif (Fig 1D).

**Fig 2:**
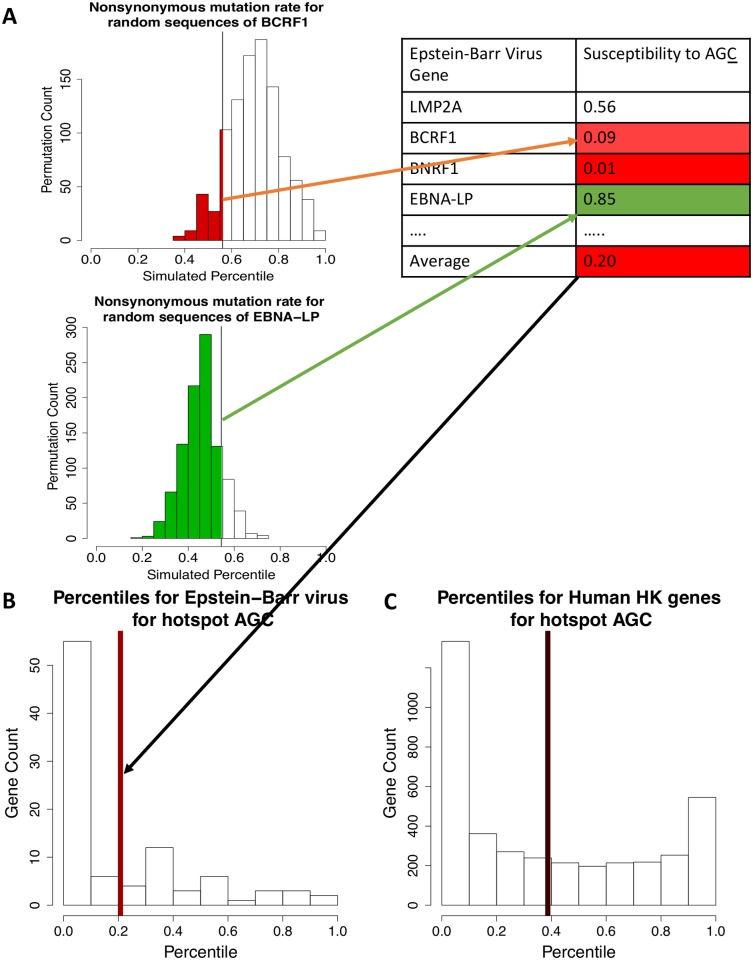
Computing average susceptibilities for each genome to a given motif. A) The susceptibility scores in the EBV genome are calculated for each gene together with the average B) Distribution of percentiles in the EBV genome and C) in the human housekeeping gene set, to the AID motif AGC. The average, shown in a bold colored line, is low (close to 0) in a very resistant genome.

We next examined how mutational susceptibility of retrotransposons and viral genomes was affected by different APOBEC preferences. We defined a gene set as a group of genes with a common biological function, for example, a set of housekeeping genes, or the genes in a particular virus. Starting with the individual susceptibility measures for each gene, we calculated the average susceptibility for each gene set (Fig 2A). As an example, Fig 2B shows the susceptibility values for the Epstein-Barr Virus (EBV), a herpesvirus that is tropic to B-cells, potentially exposing it to AID, which has a strong preference for mutating AGC motifs.

We reasoned that under a null model, if susceptibility is equivalent to a P-value, then random percentiles would assume a uniform distribution from 0 to 1, and would have a median and mean of 0.5. In contrast, the average susceptibility of the EBV genome is approximately 0.2 and for the set of human housekeeping genes it is 0.4 (Fig 2C), suggesting human housekeeping genes are slightly less resistant to the impact of AGC deamination. As each coding sequence susceptibility score can be treated as a P-value, to assess the susceptibility of each gene set statistically we combined the individual P-values using an unweighted Z-transform approach, which is more powerful than the standard Fisher method [61]. We found this approach was capable of detecting significance even for small gene sets, such as HIV or Adeno-Associated Virus 2 (AAV2), which have only 7 and 8 genes respectively but show significant results for several motifs (S1 Table). In the example of Fig 2B, the susceptibility of the EBV genome to the AGC motif was statistically lower than a null median of 0.5 (P < 10^−49^, S1 Table) and in general EBV displays statistical resistance to 13 of the 16 NNC motifs we examined (using a cutoff of P < 3.125 × 10^−3^ for significance, representing Bonferroni corrected P = 0.05). We applied the same z-score method to determine the statistical vulnerability of a gene set, defining it as the combination of the scores (1-susceptibility) to obtain a P-value (S2 Table).

### Differences in susceptibility across genomes

To quantify the impact of deamination on various genomes we analyzed a diverse set of genomes, including viruses previously reported as AID or APOBEC targets, sets of housekeeping genes from mammalian (mouse and human) genomes and, as controls, genomes from viruses of non-vertebrate hosts that have evolved in the absence of AID/APOBECs (see Methods section “Data Sources”, and Table 2). For each of these gene sets we were interested in quantifying the impact of C>T mutations at the reported hotspots for AID/APOBEC (Table 1). Since we wanted to gain insight into why these particular hotspots evolved, we considered all motifs of the form NNC (N = any nucleotide), which includes 10 hotspots targeted by AID/APOBEC and 6 hypothetical hotspots such as GCC that have not evolved specificity within the AID/APOBEC family. We measured susceptibility values (as described above) for every gene within each gene set, calculating the mean susceptibility in each case. This was repeated for all 16 NNC motifs, thus obtaining 16 susceptibility values for each gene set.

**Table 2:** Summary of gene sets analyzed.

	Established Deaminases	# of sequences	Source/Accession Number
Mouse AID Off-Targets	AID	16	See: Methods
Human Housekeeping genes	Potentially all	3804	See: Methods
Mouse Housekeeping Genes	Potentially all	2210	See: Methods
Little Cherry Virus 2	None	10	NC_005065
Strawberry chlorotic fleck associated virus	None	10	NC_008366
Ostreid Herpes Virus	None	127	AY509253.1
Human L1 Elements	hA3B	122	See: Methods
Mouse L1 Elements		99	See: Methods
Mouse MusD		3	AB305072
Human Herpesvirus 1	hA3A,hA3B	77	NC_001806
Epstein-Barr Virus	None, active in B-cells with AID present	95	NC_007605
HPV 16		8	NC_001526
AAV2	hA3A	8	AF043303
Hepatitis B Virus	All APOBECs, hA3A	7	NC_003977
Hepatitis C Virus		2	NC_004102.1
HIV	hA3G	7	L20571
Murid Herpesvirus 2		167	NC_002512
Murid Herpesvirus 68		74	NC_001826
HTLV-1	hA3G	8	NC_001436.1
TTV		4	AY823988.1
Simian Foamy Virus (SFV)		6	NC_001364
HERV (Specifically, HCML-ARV example)		3	AY208746.1

**Fig 3:**
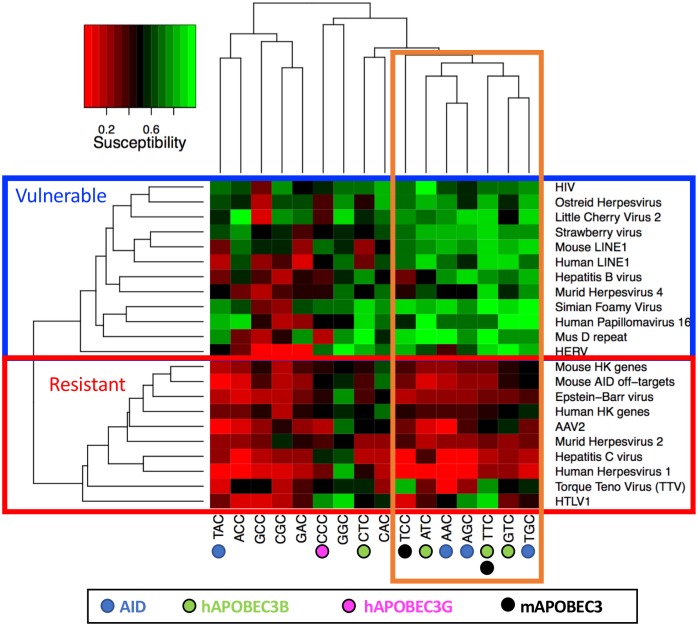
Gene sets cluster by hypothetical NNC motif susceptibility. The clustering suggests that the gene sets we examined segregate clearly into AID/APOBEC-resistant (boxed in red), and AID/APOBEC-vulnerable clusters (boxed in blue). Many of the known hotspots for AID and APOBEC also cluster together (boxed in orange).

We observed that one of these gene set clusters (indicated by the red box labeled “Resistant” in Fig 3) has high overall resistance (i.e., low susceptibility) to NNC motifs. This resistant cluster includes many viral genomes, such as Human Herpesvirus 1 [31], and hepatitis C Virus [35]. Curiously, this cluster includes the Epstein-Barr Virus, suggesting that this virus may have evolved resistance to mutations by both APOBECs and AID. Although to our knowledge no studies have demonstrated a direct mechanism of AID restricting EBV experimentally, the virus is B-cell tropic and would presumably be exposed to AID deamination in germinal centers and to an extent in extrafollicular compartments [62]. Gene sets of host (mouse and human) housekeeping genes also show overall resistance to mutations in NNC motifs. Housekeeping genes will, by definition, be co-expressed with AID and those APOBEC genes wherever they are expressed. Although little is known about the mechanisms by which our host genomes avoid APOBEC mutations, the evidence from AID in B-cells shows that mutations indeed occur genome-wide but are repaired, although the repair process is imperfect [56]. Our analysis here suggests that this mutational behavior (perhaps including APOBEC) may have created selective pressures on the genome to minimize this potentially hazardous activity.

The cluster of gene sets in the blue box of Fig 3 and labeled as Vulnerable had higher mean susceptibility scores across all NNC motifs. The cluster includes a human endogenous retrovirus (HERV), the human (LINE-1) and mouse retrotransposon (LINE-1 and MusD) coding regions, as well as the control genomes, which include viruses of invertebrate hosts (oyster, plants) that would not have co-evolved with AID or APOBEC. Surprisingly, there are several virus gene sets that are targeted by APOBEC and that we did not initially expect to be vulnerable, including HIV, Simian Foamy Virus (SFV), Hepatitis B Virus, Murid Herpesvirus 4 and Human Papillomavirus 16. HIV’s apparent vulnerability is surprising since HIV is a well-known target of human APOBEC3G. However, as described in the Introduction, HIV encodes *vif*, an APOBEC3G inhibitor, which may obviate the need for sequence-level avoidance. Other unknown defense mechanisms for these targeted viruses may also explain their apparent vulnerability. Mouse APOBEC3 is incapable of impairing MHV68 (Murid Herpesvirus 4) viral replication, for example, whereas several human APOBEC3s are capable of restriction, suggesting an alternate, unknown anti-mAPOBEC defense may be present [63]. Furthermore, recent work on HIV has shown that APOBEC3G may in fact help the virus mutate and create escape variants [64], which may in turn explain its relatively high level of susceptibility. A similar explanation may hold for human papillomavirus (we analyzed HPV16), which recent studies have shown to be extensively mutated *in vivo* and *in vitro* [28]. In light of these phenomena, our results suggest that even if a virus targeted by APOBEC is found to be susceptible to APOBEC hotspots, the virus itself may contain an alternate method of escape such as *vif*, subversion of APOBEC for evolutionary purposes, or other factors.

Considering now the hierarchical clustering results for the 16 NNC motifs (vertical dendrogram of Fig 3) we observed that many existing (or “canonical”) AID/APOBEC hotspots, including 7 of the 10 AID/APOBEC hotspots are contained within the same cluster (right-most 7 motifs in Fig 3, boxed in orange). This cluster contains 3 of the 4 canonical AID hotspots AGC, AAC, and TGC (only excluding TAC), as well as 3 of the canonical human APOBEC3B NTC hotspots (ATC, GTC, and TTC, only excluding CTC), and the mouse APOBEC3 hotspot TCC, but excludes the human APOBEC3G hotspot CCC. In light of the contrast observed, the existence of the two clusters, resistant and vulnerable, suggests that AID/APOBEC hotspots cause high mutational damage to exogenous viruses and native transposable elements. At the same time, we have observed that many of the genes targeted (intentionally in the cases of viruses and unintentionally in the case of host housekeeping genes) show resilience to the impact of these mutations at hotspots. This resilience may have evolved as an avoidance strategy to minimize mutations if the hotspots that APOBEC evolved do not cause high collateral damage to the host genome.

### Alternative models and controls corroborate observed susceptibilities

GC bias is an important evolutionary constraint in many viruses, and may play important APOBEC-independent roles such as protection from insertions [65]. Although we demonstrate later that the GC content of the gene sets considered correlates strongly with susceptibility (see next section “GC content of genomes predicts deaminase hotspot susceptibility”), we sought to quantify the extent to which native GC content contributed to the observed susceptibilities. To that end, for each of the gene sets considered, we generated new susceptibility scores that controlled for the GC content by defining a new null model that used the GC content of the gene set being analyzed to select codons (see Methods). Using the same unweighted z-score calculation as described above to determine statistical significance, we observed that for the high GC-content EBV genome, 9 out of the 16 NNC trinucleotide contexts that we examined were still statistically resistant (compared to 13 out of 16 in the uncorrected case). For the vulnerable OsHV genome, 6 out of 16 NNC trinucleotides were statistically vulnerable (compared to 10 out of 16 in the uncorrected case). We confirmed these trends again by recalculating susceptibilities for each of the gene sets, now using the GC-corrected null model (S2 and S3 Tables). We visualize these trends with another heatmap similar to Fig 3 which maintains the ordering of the columns and rows for ease of comparison (S1B Fig). Although the trends in susceptibility are similar to the non-GC-corrected case, the differences between vulnerable and resistant gene sets are reduced, suggesting that GC content explains a great deal, though not all, of the observed resistance to APOBECs.

Additionally, there is a question of whether the total number of nonsynonymous mutations should be minimized, or whether it is more appropriate to examine the nonsynonymous mutation fraction, which normalizes the number of motifs. There are two possible scenarios to consider for the frequency of mutation. In one scenario, a gene may only be exposed to the mutagen a few times during its activity. This is observed in B-cells, where AID is exposed to a gene possibly only once per cell cycle [8]. In this scenario, because the number of mutations is limiting, the probability that a mutagenic event causes an amino acid change is reduced by minimizing the *fraction* of nonsynonymous mutations, i.e. the number of nonsynonymous mutations divided by the total number of motifs. If a mutagen is exposed to a sequence only rarely, as in the case of AID, a coding sequence could minimize the probability of each mutagen changing its sequence by having many hotspots in the silent position. In the second scenario, mutations are not rate-limiting and the absolute number of hotspots causing nonsynonymous mutations would need to be minimized rather than the fraction. We found that tests conducted under the second scenario show similar results, albeit with slightly weaker trends (see section below “GC content of genomes predicts deaminase hotspot susceptibility“). Thus, our results are clearest in the first scenario (fraction of nonsynonymous mutations) assuming it is more indicative of susceptibility to mutagenesis. We conclude that examining the fraction is more instructive in determining trends (S2A Fig).

Additionally, as a control, we applied this model to different mutation profiles rather than the standard C to T. We chose every type of transition mutation (NNA to NNG, NNT to NNC, and NNG to NNA, which is complementary to NNC to NNT). In the cases of NNA to NNG and NNT to NNC (S2B and S2C Fig), we observe trends that are the opposite of our findings as described in the main text. Averaged across all 16 hypothetical NNA -> NNG hotspots, the susceptibility is lower in the oyster herpes virus by 0.18 (P = 0.011, 2 tailed t-test) relative to the housekeeping genes, though still prevalent but not as strong for the L1 elements (difference = 0.10, P = 0.24). Similarly, with all 16 NNT to NNC T to C mutations, L1 (difference = 0.12, P = 0.048) and oyster herpes viruses (difference = 0.198, P = 0.0063) show lower susceptibility to hotspots relative to human housekeeping genes. This suggests that mutagenesis at NNC to NNT hotspots is preferable for host genomes compared to these other patterns of mutation. We applied the same analysis as we did in Fig 3, to the hypothetical mutations of NNG to NNA (S2D Fig). Because we consider motifs on both strands, NNG to NNA is equivalent to CNN to TNN. For NNG to NNA we observed some similarity to the NNC to NNT case. Again there appear to be groups of resistant and vulnerable gene sets and these overlap somewhat with the NNC to NNT case, although the clusters are less clearly separated. If the average susceptibility of each gene set (rows in Figs 3 and S2D) were the key feature separating the vulnerable and resistant clusters, then we would expect similar clusters for both NNC to NNT and CNN to TNN. However, the clusters in each case are not the same suggesting that hotspot (column-) specific features that are unique to each cluster, are also important.

Although considering all 12 possible types of mutation is beyond the scope of this study, we did consider the three transition mutations other than NNC to NNT (S2B–S2D Fig) and one transversion (NNC to NNG, S2E Fig) that is directly comparable to Fig 3. Concerning the latter case, we found some similarities to the NNC to NNT transition case in that all control and retrotransposon gene sets clustered together in a predominantly vulnerable group. Clearly, some similarity is to be expected since most mutations at the third position of a codon are synonymous, and the NNC contexts are shared between the two cases.

### GC content of genomes predicts deaminase hotspot susceptibility

When we analyzed susceptibilities using the total number of nonsynonymous replacements rather than the fraction of replacements, we observed greater variability across the 16 NNC motifs (S2A Fig). In spite of this, at the highest level the same two clusters of vulnerable and resistant gene sets emerge. However, under this assumption many of the gene sets in the resistant cluster, such as the mammalian housekeeping genes and the Epstein-Barr virus genome here show high vulnerability to GC rich motifs. These motifs include the APOBEC3G hotspot CCC, and the hypothetical hotspots GCC and GGC, all three of which cluster together. These gene sets simultaneously show resistance to the AT rich motifs ATC, TTC, TAC, and AAC, which are also clustered together. Varying GC content of the gene sets may explain these discrepancies. A gene with a very high GC content would contain more GC-rich motifs and fewer AT-rich motifs (and therefore a higher raw nonsynonymous mutation count) by chance. However, it is surprising that the trends are similar to our observed susceptibilities (Fig 3), since our definition of susceptibility examines only the nonsynonymous mutation fraction instead of raw count, “normalizing” for the GC content. Our analysis from here on thus examines the model of susceptibility that calculates the percentile of nonsynonymous mutation *fraction* compared to random sequences.

**Fig 4:**
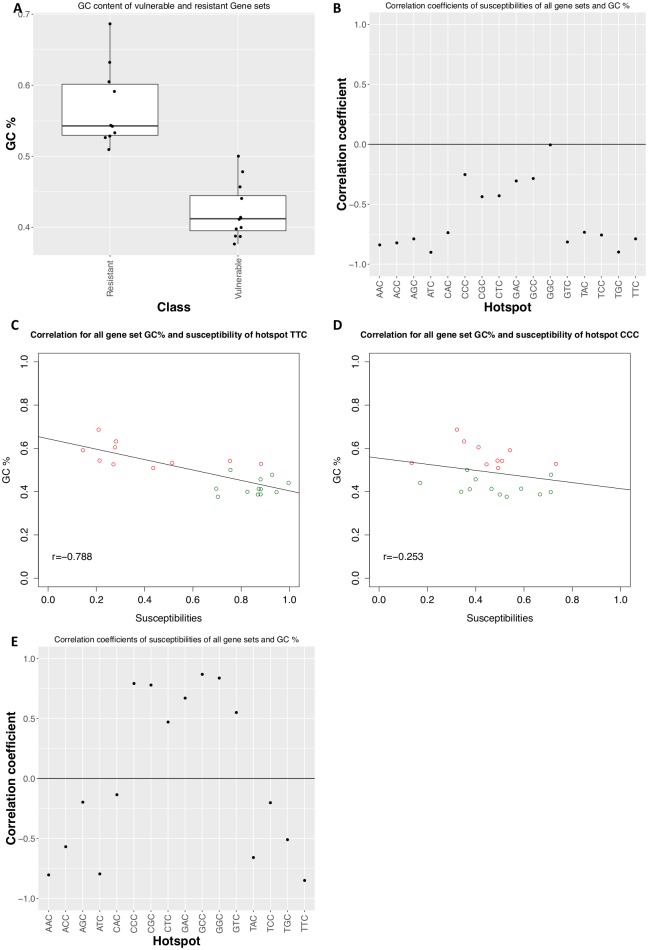
Low GC content is correlated to susceptibility to some NNC motifs. A) Boxplots show the distribution of GC content of APOBEC-resistant (left) and APOBEC-vulnerable (right) gene sets. B) Plot showing the correlation coefficient (Y-axis) between our gene sets’ susceptibility to a particular motif on the bottom axis to its GC content. C) The correlation of a gene set’s mean susceptibility to the hotspot TTC, a GC-rich hotspot, to its GC content (red = resistant, green = vulnerable based on our clustering observations). D) Correlation of a gene set’s GC content to mean susceptibility for the hotspot CCC. E) Correlation between GC content of all gene sets and a measure of susceptibility based on the absolute number of nonsynonymous mutations at motifs. Susceptibility in this case is negatively correlated with motifs beginning with A and T, and more positively correlated with motifs beginning with G and C.

It is not immediately clear how GC content will also affect the nonsynonymous mutation fraction that is used to calculate susceptibility (Fig 3). However, a gene set with varying GC content may adopt a codon usage that inherently favors APOBEC hotspots at positions that cause synonymous mutations. We therefore compared the GC content of our vulnerable and resistant gene sets and found a strong statistical difference, with resistant gene sets having much higher GC content (Fig 4A, 2-tailed t-test, P <1.0^−5^*)*. Furthermore, we observed that the correlation of susceptibility with GC content was strong for some motifs and not others. For many of the 16 motifs we examined, a higher GC content correlated strongly with low susceptibility to that motif as demonstrated by very negative correlation coefficients (Fig 4B). For example, the AT-rich hotspot TTC showed a strong correlation (Fig 4C), but for the GC-rich hotspot CCC the trend is not as apparent (Fig 4D). There is zero correlation between susceptibility of the motif GGC (which is not an observed AID/APOBEC hotspot) and GC content. Finally, to further corroborate these trends we have assessed the correlations between GC content and the alternate definition of motif susceptibility which considers the absolute number of nonsynonymous mutations rather the fraction. We observe again that a low GC content confers stronger susceptibility (negative correlation) to motifs of the WNC (W = A/T, N = any nucleotide) motif (Fig 4E), consistent with our findings to be discussed below (see section: “Retrotransposons and viruses show great discrepancy in resilience to biological deaminase hotspots.”). Our analysis suggests that GC content is strongly predictive of existing APOBEC hotspot susceptibility, with the exception of the human APOBEC3G hotspot CCC.

### Differences in mutational disruption of retrotransposons and viruses arising from canonical deaminase hotspots

The results shown in Fig 3 suggested that the current APOBEC hotspot preferences may have evolved to mutate particular targets while minimizing self-damage. To explore this further, we asked which of the 16 possible NNC motifs were best in discriminating host against parasitic (viral, transposable element) genomes. We proceeded by performing all pairwise comparisons for each gene set in the resistant cluster to each gene set in the vulnerable cluster, noting the differences in the susceptibility scores for each of the 16 NNC motifs. Since we observed 10 resistant gene sets and 12 vulnerable gene sets (Fig 3), this resulted in 120 individual comparisons. For each comparison the 16 NNC motifs were sorted by the difference in susceptibility of the resistant to the vulnerable genome (resistant—vulnerable). As an example, when we compared human housekeeping genes (resistant) to L1 retrotransposon elements (vulnerable) we discovered that the housekeeping gene sets had an average susceptibility to the motif ATC of 0.39, whereas the average susceptibility for the L1 elements was 0.872 (S4 Table). Thus, with a difference of -0.482, the APOBEC3A/B hotspot ATC shows the strongest contrast out of any of the 16 motifs and is ranked the highest, whereas the motif GAC has a difference of +0.321 and is ranked the lowest. The rankings suggest that ATC, rather than GAC, might evolve to be a preferable motif due to its capability for damaging L1 ORFs more so than impacting host genes.

**Fig 5:**
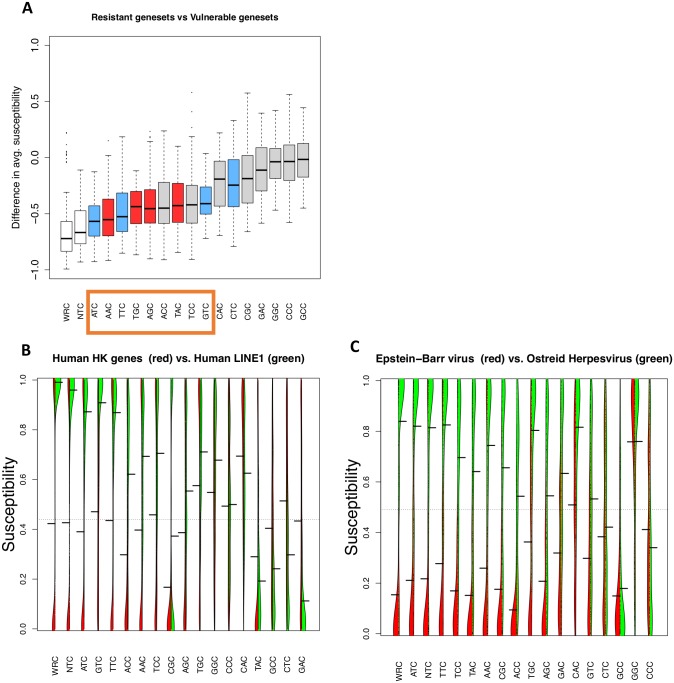
The naturally observed cytidine deaminase hotspots better distinguish vulnerable and resistant gene sets than hypothetical NNC motifs. A) Boxplot distribution of differences in average susceptibilities between APOBEC-vulnerable and APOBEC-resistant gene sets. Canonical AID hotspots are in red, and APOBEC3A/B hotspots are in blue. The degenerate motifs WRC and NTC, are labeled in white. The top cluster of motifs (boxed in orange) are enriched in these motifs. B, C) Violin plots of separate gene families’ susceptiblities, clustered (left family in red, right in green), with protruding black lines representing the mean for that gene set. Motifs are sorted by the greatest difference in mean susceptibilities, with many of the motifs showing the greatest difference having the motif NTC or WWC (W = A/T).

By analyzing all 16 motifs across 120 comparisons, we found that the rankings of human APOBEC3B (NTC, N = any nucleotide) and AID (WRC, W = A/T, R = A/G) hotspots in particular, were significantly lower than expected by chance (see Methods, bootstrapped P = 0.0071 and P = 0.0002 respectively). We further calculated, for each motif, the average difference across all pairwise comparisons of vulnerable and resistant gene sets, again ranking each motif by the overall difference in susceptibility. For 9 out of the 16 motifs we examined, the susceptibility of the vulnerable genes was strongly lower than that of the resistant gene sets to a similar degree, a difference that was highly significant (t-test, P < 2.2 × 10^−16^ for all 9 motifs, Fig 5A columns shown in orange box). Curiously, these 9 motifs which demonstrate the greatest difference in mean susceptibilities also include all 8 motifs defined by the motif WNC (W = A/T). Although WNC itself is not necessarily a canonical preference seen in an existing cytidine deaminase, this set of 9 significant motifs still contains considerable overlap with existing cytidine deaminases, including all AID hotspots (WRC, where R = A/G), three of the four possible APOBEC3A/B hotspots (ATC, TTC and GTC), the human APOBEC3C hotspot (TTC) and the murine APOBEC3 hotspot (TCC).

As an alternative method to confirm the significantly lower susceptibility of these 9 motifs (WNC and GTC), we compared the average rank of the 16 NNC motifs across all of our 120 pairwise comparisons, noting that the average rank of the 9 motifs were all statistically lower than expected by chance, whereas the average ranks of the other 7 motifs were higher than this (S5 Table, BH-corrected P < 10^−4^, one-sample MWU). Additionally, although it was not possible to include degenerate motifs such as WRC and NTC in our original analysis due to the non-independence with other observed motifs such as ATC, we did calculate susceptibility scores for these two motifs separately, as described next.

The 9 motifs we identified here as having lower susceptibility (Fig 5A) includes all 7 motifs that we previously indicated as distinguishing APOBEC-resistant and vulnerable gene sets based on the clustering of motif susceptibility (Fig 3, orange box). These 7 motifs cluster primarily as a result of their biological ability to distinguish vulnerable and resistant gene sets. Additionally, the two degenerate motifs of respectively AID (WRC) and hAPOBEC3B (NTC) showed large differences between vulnerable and resistant motifs as well (shown in white in Fig 5A). Both results suggest that the motifs that most strongly distinguish APOBEC-resistant and vulnerable genomes tend to be the existing hotspots, rather than hypothetical hotspots not associated with AID or APOBEC.

We observed that broadly, the vulnerable cluster (Fig 3, blue box) contains two qualitatively distinct families of genes, notably viruses and transposable element clusters. Similarly, the resistant cluster includes endogenous host genes such as the housekeeping gene set, and also viruses such as EBV and HSV-1, that have likely co-evolved with the APOBECs and may have acquired low APOBEC susceptibility as a consequence of this co-evolution. We assumed that the preferred APOBEC hotspots would be those that maximally damage their intended targets in intrinsic immunity, i.e. retrotransposons and vulnerable viruses, while minimizing damage to the host genome. With this assumption in mind, we looked more closely at two particular comparisons relevant to APOBEC evolution in humans. We first examined human housekeeping genes against human L1 elements, as the L1 elements are a major biologically significant target for APOBECs [39, 66]. Secondly, we compared two viruses: the Epstein-Barr virus (Human Herpesvirus 4) from the resistant cluster, against the Ostreid Herpesvirus, which we used as a control genome since its host is invertebrate and therefore not expressing any APOBEC genes, and is categorized in the vulnerable cluster.

In the first comparison (Human housekeeping vs Human L1) we found that the top 3 motifs in terms of susceptibility differences are all APOBEC3B TC hotspots: ATC, GTC, and TTC (Fig 5B, S4 Table). Interestingly, the observed difference does not arise because human housekeeping genes are particularly resistant to these motifs, since they have mean susceptibilities of 0.39, 0.51, and 0.43 respectively, which is close to that expected by chance (0.5). However, retrotransposons are particularly susceptible to these hotspots, with susceptibilities ranging from 0.87 to 0.91 for these three hotspots, where 1 represents maximum susceptibility. These high values suggest that human APOBEC3B hotspots may have evolved due to a particularly high capability of inducing nonsynonymous mutations in L1 elements, rather than low susceptibility to these hotspots in the host genome. We verified this effect for the AID hotspot WRC on some human non-housekeeping genes as well, by comparing the susceptibilities of our human housekeeping genes to a set of B-cell specific genes [67]. We used the list of B-cell specific genes listed in this study to obtain their coding sequences from the UCSC genome browser and calculated their susceptibilities, using the longest sequence if multiple isoforms were available for a single gene. For the B-cell specific genes, the susceptibilities to the 4 WRC hotspots were even lower than housekeeping genes: (AGC: difference = 0.10, P = 0.0075), (AAC: difference = 0.1, P = 0.000038), (TAC: difference = 0.12, P = 8.8 × 10^−9^), (TTC: difference = 0.07, P = 0.00313), suggesting that B-cell genes are under even more pressure to avoid deleterious mutations at AID hotspots than other genes.

In the second case (Fig 5C, S6 Table), we compared the APOBEC-resistant Epstein-Barr virus (EBV) to the Ostreid Herpesvirus (OsHV). The most highly ranked motifs in terms of susceptibility differences again included many A/T-rich motifs, with 8 out of the 9 top motifs again having the motif WNC. Furthermore, the two motifs showing the strongest difference are the hotspots ATC and TTC (collectively the motif WTC), which are human APOBEC3A/APOBEC3B hotspots. Many of the AID hotspots that EBV might be expected to evolve resistance to (given that EBV is a B-cell tropic virus) also show strong contrast, with the hotspots TAC and AAC ranked 4^th^ and 5^th^ and TGC and AGC being ranked 8^th^ and 9^th^ respectively. These differences, in contrast to the comparison between housekeeping genes and L1 elements, arise not only due to the high susceptibility of OSHV to motifs such as ATC (0.82) and TTC (0.83), but also due to EBV’s strong resistance, i.e. low susceptibility (0.21 for ATC, 0.277 for TTC). These low susceptibilities to APOBEC hotspots in EBV, together with our observation that AID/APOBEC hotspots again show the strongest contrast between resistant and vulnerable viruses, suggest that viral genomes such as EBV have adopted resistance to this particular form of mutagenesis.

In general, we note that motifs of the form WNC, and more specifically, WTC (W = A/T) appear to show the largest difference in vulnerabilities between housekeeping genes and retrotransposons, as well as between resistant virus and vulnerable genes. While WNC itself is not necessarily a canonical AID or APOBEC hotspot, it is a superset of the well-characterized AID hotspot WRC [1]. Furthermore, WTC overlaps with the hotspot NTC seen in human APOBEC3A/APOBEC3B. Thus, the fact that many of the motifs with the largest differences correspond to existing AID/APOBEC hotspots supports a hypothesis that the ability of AID/APOBECs to act effectively against viruses and retrotransposons while avoiding damage to the host is an evolutionary advantage for the host.

### GC content and codon bias of genomes contribute to hotspot susceptibility

Many factors play an important role in shaping virus nucleotide evolution [68, 69]. We next describe how we investigated in particular the effects of codon bias, GC content, CpG dinucleotide frequency, selective pressure, and AID hotspot targeting of a hypothetical or actual virus sequence to validate our model.

We constructed new gene sets of 250 genes of completely random open reading frames that are 1500 nucleotides long, beginning with the methionine codon, ending with a stop codon, and with random non-stop codons in between. For the random codons, each nucleotide was chosen randomly, with either 40%, 50%, or 60% GC content. We calculated the susceptibility scores of these random sequences to validate susceptibility independent of evolutionary effects. Under uniform distribution of C and G (50% GC content), meaning each of the four bases is equally likely to be chosen, we observe that the median susceptibility to the canonical hotspots NTC (hA3B), CCC (hA3G), and WRC (AID) is 0.5 (S3A Fig). With random sequences of varying GC content, however, the susceptibility changes. Randomized sequences of low (40%) GC content showed high susceptibility to the hotspots NTC and WRC, and similarly, high GC content sequences showed low susceptibility (60%). We reaffirmed this by creating new randomized sequences of the resistant, high-GC content EBV and vulnerable, low-GC content OsHV, replacing each codon in each sequence with a synonymous one weighted by the GC parameter (a similar weighing description is given in the Methods under “Calculating Susceptibility”, though here it is applied to the baseline sequences rather than the null model) and observed similar trends, suggesting a strong role of wildtype GC content in explaining APOBEC resistance. We note, however, that this effect is not present for the hotspot CCC. We corroborated these effects by looking at the effect of GC content on codons for existing protein sequences. To determine the effects of native GC content on determining APOBEC hotspot resistance, we additionally calculated susceptibility under a GC-controlled null model (see Methods), weighing the GC parameter to be the mean GC content of the entire gene set. This meant that each gene sequence would now be compared to random synonymous sequences with a chosen GC content. Using this model, we found that changing the GC content of the random sequences affects the observed susceptibilities, reaffirming the importance of GC content in determining susceptibility. If a baseline (wild type) coding sequence is being compared to random sequences with higher GC content, the baseline sequence will tend to show higher susceptibilities to NTC and WRC hotspots (suggesting that lower GC content is more vulnerable to deamination). Despite these trends, the vulnerable OsHV sequence still has higher susceptibilities to NTC and WRC than EBV when GC is corrected (S3B Fig), although are not significantly so (NTC P-val: 0.18, WRC P-val: 0.34, BH corrected t-test). L1 elements, however, have their apparent high susceptibilities to NTC (the hotspot that hAPOBEC3B has which restricts them) neutralized when corrected for in this manner. Thus, high impact of these hotspots on L1 may be a beneficial effect of their inherent low GC content. There is little change in the susceptibilities of HIV to the NTC and WRC degenerate motifs (NTC P-val: 0.93, WRC P-val: 0.82) when we apply this correction, suggesting that while low GC genomes can be resistant to AID/APOBEC hotspots, there can be genomes for which their GC content does not contribute strongly to their vulnerability.

Additionally, we obtained codon usage tables of the coding sequences of OsHV, EBV, and our human housekeeping genes and L1 elements using Emboss cusp (http://www.bioinformatics.nl/cgi-bin/emboss/cusp). When we compared OsHV to EBV, we regenerated sequences in each genome using the codon usage table of the other and calculated the susceptibility of the new sequence. We observed that OsHV, when shuffled with codons with probability proportional to the codon usage bias of EBV, adopts a susceptibility much closer to that of EBV, and vice versa (S3C Fig). We observe similar effects when we exchange the codon biases of HK and L1 elements (two mammalian gene sets that we assume would be differentially targeted by APOBECs) and calculate susceptibilities (S3C Fig). We conclude that the nature of APOBEC hotspot susceptibility is also influenced by codon bias for both virus and vertebrate genes. However, note that the results described above using the GC-corrected model in effect creates a model with codon bias according to wildtype GC content. Thus, it is most likely that our results here using codon bias changes (S3C Fig) are correlated with those using GC-content changes (S3B Fig).

Additionally, codons within the same amino acid can be classified as “intra-codon” or “inter-codon” depending on whether they are a NNC with C to T at the silent position or not. For example, AGC is an intra-codon for serine, since AGC to AGT does not change its amino acid, but AGA would be an inter-codon. Statistical tests of all intra- and inter-codons comparing the vulnerable OsHV and resistant EBV show that OsHV has statistically fewer intra-codons than EBV (difference = 0.182, Fisher Test P < 2.2 × 10^−16^, 2-tailed Fisher test), providing additional evidence for the role of codon bias in hotspot susceptibility. Finally, our findings on codon bias were corroborated by the trinucleotide distribution of resistant and vulnerable gene sets (“Clustering algorithm”, under Methods).

Selective pressure was also modeled by taking the original EBV sequence, a resistant genome, and inducing mutations at different positions with probability weighed to either favor nonsynonyous or synonymous mutations, according to a parameter we use to approximate the dN/dS ratio. dN/dS represents the ratio of the number of observed mutations at nonsynonymous sites per site, to the number of mutations at synonymous sites per site. Specifically, mutations occurring in the first two positions of a random codon almost always represent nonsynonymous mutations and mutations in the third position are typically (but not always) synonymous. Therefore, we mutated positions along the EBV sequence with probability weighed by a parameter. This parameter is the ratio of mutation at the first two positions to the third, and acts under the assumption that each site is equally likely to be mutated and that this ratio of mutations is correlated to dN/dS. For example, if this parameter is equal to 2, then a mutation at each position of the codon is equally likely, since 2/3 of the mutations would be nonsynonymous, affecting one of the first two positions, and the remaining mutations would affect the third nucleotide, which is often synonymous. The corresponding value for the dN/dS ratio would be closer to 1, as there are twice as many nonsynonymous sites as synonymous. Under these assumptions, dN/dS is roughly half of our selective pressure parameter. For values of this parameter being 0.25 (red), 1 (green), 4 (blue), and 8 (purple) and for up to 500 mutations over time we calculated susceptibilities for all 16 NNC motifs (S3D Fig). Over time, averaged across all these motifs, the low susceptibility changes to a higher susceptibility, which may be explained by a change in GC content to neutral (or 50%) as each of the 12 mutation types (from one base to another) are equally likely. If dN/dS is high, however, the change is smaller. Indeed, the susceptibilities between the observed EBV sequences and EBV after 500 mutations when our selective pressure parameter = 8 (approx. dN/dS = 4) is not statistically significant except for the motifs GCC and GGC (GCC: P = 0.0004694, GGC: P = 0.0053; G2-tail t-test, BH corrected). The difference however between EBV after 500 mutations under high selective pressure (parameter = 8, approx. dN/dS = 4) and low (parameter = 0.25, approx. dN/dS = 0.125) is significant for many motifs including AAC (P = 0.0019), ACC (P = 0.0018), ATC (P = 0.0018), CGC (P = 0.0019), GAC (P = 0.025), TAC (P = 0.00039), TCC (P = 0.0377), and TTC (P = 0.0018). Based on our simulations, high selective pressure appears to play a modest role in maintaining APOBEC hotspot resistance. We note that many mammalian herpesviruses display resistance (Fig 3), and if APOBEC deamination is deleterious to survival of the virus, then there would be evolutionary pressure on the virus to change in such an environment. However, this may be mediated simply by changing the GC content of the virus.

Finally, there are other models of APOBEC/AID activity at hotspots. One of these is the S5F model of Yaari et al. [70] which inferred the mutability and substitution profile during somatic hypermutation among all 1024 possible 5-mers that targeted silent positions and which are therefore assumed to be independent of the effects of selection. Although somatic hypermutation includes not only AID activity, but also non-canonical Base Excision Repair and Mismatch Repair, the model is arguably the most comprehensive available for somatic hypermutation. To investigate the impacts of this activity on a gene’s susceptibility over time, we used this model and induce mutations at each position to the vulnerable OsHV and HIV genomes, resistant EBV genome, and random sequences, with probabilities proportional to the S5F model. This model includes the effects of base excision and mismatch repair which induce mutations beyond that which occur at hotspots, so we also simulate mutations using the same frequencies but only at C to T. We induce a number of mutations equal to up to 60% of the sequence length (to account for different gene lengths). Our results show that when we consider susceptibility to the AID hotspot WRC, AID targeting decreases susceptibility to WRC motifs (S4A Fig), highlighting the possibility that mutations at non-hotspot sites can decrease the susceptibility of a gene. In the case where we apply S5F, but only mutating C to T (and thus also G to A on the opposite strand), we mutate up to 60% of C and G positions instead per gene. Under this mutational model, that ignores the DNA repair mechanisms that act downstream of AID, we observe the opposite trend, namely that susceptibilities to the WRC motif instead increase as mutations accumulate (S4B Fig).

To extend this analysis beyond AID and somatic hypermutation, we also modeled the activity of two other APOBECs. We performed a simple simulation of APOBEC mutation at TC (hAPOBEC3B) and CCC (hAPOBEC3G) hotspots and then calculated the changes in susceptibility to those motifs accordingly. When we mutate the resistant EBV sequence at the hotspot NTC, susceptibility increases to a high level (S4C Fig). Interestingly, we did not see a change in susceptibility to the other hotspot CCC (S4D Fig) for all genomes that we examined. This suggests that regardless of the number of hotspots that are at nonsynonymous or synonymous positions, susceptibility to hotspots can be altered by GC content. The fact that CCC susceptibility, a hotspot which appears neutral to GC content (S3A Fig), does not change by this mechanism lends support for this idea.

Finally, the CpG dinucleotide has biological significance as a marker for DNA methylation and therefore gene activation and inactivation, especially in mammals [71]. We calculate another susceptibility measure that properly controls for this frequency (See: “Controlling for CpG dinucleotide content” under Methods). As shown in S5 Fig, when we consider this, most of the gene sets clustered similarly to the uncorrected method (Fig 3). As far as clustering of the motifs is concerned, we obtained one cluster of 6 motifs (rightmost dendrogram of S5 Fig), containing all 4 ANC motifs including two AID hotspots, AGC and AAC, which strongly overlaps with the cluster of motifs obtained without correcting for CpG (as described above for Fig 3, which contains 3 of the 4 ANC motifs, 3 of 4 TNC motifs, and GTC). These results suggest that the impact of these motifs is still similar when measuring susceptibility controlled for CpG. The gene set clusters also remain the same as the clusters without controlling for CpG content, with the exception of two formerly vulnerable gene sets, MHV68 and HERV which now cluster within the resistant gene sets. These gene sets, respectively a mammalian virus and set of retrotransposons, are co-expressed in environments alongside APOBECs, and in the case of HERV, is strongly edited by them [24]. Since accounting for CpG does not appear to affect the results very strongly, we do not control for this motif in the analyses, although this observation suggests that adapting CpG usage can be a mechanism of reducing effective susceptibility for a subset of viruses, particularly HERV.

Altogether our results suggest that resistance to APOBEC hotspots is a natural consequence of GC content, and provides an explanation of how, under selective pressure, a coding sequence may adopt resistance to APOBECs. These findings are consistent with the results described above (Fig 4) where we found a strong correlation between a genome’s GC content and its motif susceptibility. High GC content, for example, is a feature of several herpesviruses, and a previous study proposes that GC content may play a previous protective role against retrotransposon insertion in these viruses [65]. While we are not suggesting that gene sequences always adopt or evolve their GC content solely for the purposes of APOBEC resistance, we do show that GC content adjustment may confer additional defense against the consequences of restriction by most APOBECs, with the notable exception of the hAPOBEC3G hotspot CCC.

## Discussion

We have introduced a method of quantifying the susceptibility of gene coding sequences to mutational hotspots. We used this method to examine differences in susceptibilities across many host and pathogen genomes and tested both known and hypothetical cytidine deaminase targets. We observed that the known hotspots, particularly the APOBEC hotspots, have a high capacity for inducing non-synonymous mutations in retrotransposons and viruses, while minimizing damage to host genes. Although it needs further investigation, our data suggests a possibility that this selectiveness has been important in shaping AID/APOBEC hotspot preferences during vertebrate evolution.

Susceptibility is potentially influenced by many non-independent factors, including GC content, amino acid usage, and codon bias. Our basic measure of susceptibility is neutral to GC content and codon bias, allowing us to quantify the extent that these factors can evolve to influence susceptibility and its counterpart, resistance. In some cases GC content, via its effects on codon usage, strongly influences susceptibility, while in other cases the effect is less clear (Fig 4A–4D). We expect that a C/G rich hotspot such as the APOBEC3G hotspot CCC should be correlated to the genome’s GC content and indeed, many GC-rich herpesviruses such as HSV-1 (68% GC) and EBV (60% GC) show high susceptibility to CCC (as well as to the hypothetical hotspots GGC and GCC) when we use an alternate definition of susceptibility which considers the absolute number of nonsynonymous C to T mutations, rather than the fraction (S2A Fig). However, the situation is complex since we found cases where GC-rich genomes showed resistance to even GC-rich motifs such as CGC (S2A Fig). We conclude that evolving the optimal fraction of nonsynonymous motifs is more important than the absolute number in order to resist a broad variety of APOBEC mutations, since it is the fraction that is minimized across the resistant gene sets. Alternatively, the motif count (as opposed to the fraction) may still play a role in achieving susceptibility and it is possible a virus may evolve a high GC content to reduce the potential damage of A/T rich hotspots such as TC (human APOBEC3A/APOBEC3B) and TTC (human APOBEC3F). Additionally, tetranucleotide motifs for APOBEC3F and APOBEC3G have been observed [41] where the +1 position is also important, and although we have done the analysis for NNC motifs only, future work might include more complex motifs as well.

We have controlled for the frequency of the CpG motif (see under Methods: “Controlling for CpG dinucleotide content”, S5 Fig), however an extended analysis (that we leave for future work) might also consider the possibility that resistance is a consequence of conservation of the CpG motif itself. CpG sites in vertebrate hosts will often be methylated and are prone to spontaneous deamination. At particular loci, deamination of CpGs may be disadvantageous and evolutionarily selected against. Interestingly, AID and some APOBEC enzymes including APOBEC1can deaminate methylated Cs [4]. Thus, there may be some interplay between spontaneous and AID/APOBEC mediated deamination of methylated CpG sites. Given the additional constraints imposed by the coding region sequence, this interplay is potentially quite complex, and we therefore leave the corresponding analysis for future work.

A high GC content appears to confer resistance to deamination by many of the naturally occurring cytidine deaminases (including hAPOBEC3B, characterized by the hotspot TC and AID, characterized by the hotspot WRC). Additionally, the diversification of APOBEC3 hotspots observed in primates may favor a wider spectrum of intrinsic immune responses, as we have shown that by the same metric, genomes with high GC content remain vulnerable to hAPOBEC3G’s CCC hotspot. However, even when accounting for the endogenous GC content of the gene sets considered, we observed similar, albeit less significant overall trends (S3 Table). Thus we have shown here that there is an advantage to having a high GC content in that it tends to minimize the impact of most APOBEC hotspots. In the future deaminase resistance might also be validated experimentally, for example, by examining the viral fitness of vulnerable or low-GC content herpesviruses in APOBEC-deficient cell lines.

One of our important observations was that retrotransposon ORFs from the L1 families had very high susceptibility scores, with some having average susceptibility as high as 0.9. As our definition of susceptibility is based on the fraction of mutations that cause amino acid changes, one potential explanation for high susceptibility is that past mutations in hotspots have more frequently caused synonymous mutations rather than nonsynonymous ones. This bias would (as expected) cause the remaining hotspots to become enriched for potential nonsynonymous mutations. Also, it would be incompatible with the observation that APOBEC restriction of retrotransposons is usually deaminase-independent, as has been shown in cell culture experiments [72]. However, this observation is in dispute, as *in vivo* and *in* vitro assays have shown that hAPOBEC3A can deaminate exposed single-stranded LINE1 DNA [73]. Recent results show that older retrotransposons are often heavily edited for deactivation and that high retrotransposon editing can be beneficial in terms of genome diversification and potential exaptation [66, 74]. Additionally, a recently published study provides phylogenetic and experimental evidence suggesting that even throughout primate APOBEC3A evolution, L1 elements were unable to evolve resistance to APOBEC3A restriction, which may explain our high observed susceptibilities of L1 ORFs to APOBEC3 hotspots [75]. Thus, a high L1 ORF hotspot susceptibility may be beneficial to the host by driving exaptation and new functionality.

One possible implication that arises from our results is that the evolved hotspot preferences may have in turn shaped the genomes of viruses that target those cell types where the deaminases are expressed, suggesting a possible arms race (past or ongoing) between viruses and the vertebrate intrinsic immune systems. It is important to note that while the susceptibility measure may reflect the evolutionary impact of deaminases, it might not always implicate AID/APOBEC as an active agent. The low susceptibilities to canonical hotspots that we have observed in virus genomes that are likely to have ongoing exposure to AID/APOBEC, such as EBV and HSV-1 (Fig 3) suggest that this feature may be evolutionarily advantageous.

We observed that HIV was a vulnerable gene set with high susceptibilities, yet the HIV *vif* protein neutralizes APOBEC3G, targeting it for degradation, which may abrogate the need to evolve resistance at the genome level. Similarly, another herpesvirus we examined, MHV 68 (a strain of Murid Herpesvirus 4), showed high susceptibility to APOBEC hotspots including mouse APOBEC3 hotspots defined by the motif TTC. A recent study showed that the mouse APOBEC3 does not restrict this virus either in cell culture or *in vivo [63]*, suggesting the possible existence of an as yet unidentified APOBEC evasion mechanism. MHV68, alongside the transposon-like HERV elements, also show reduced susceptibility when we control for CpG dinucleotide motif frequency (S5 Fig). Thus, for some genomes the CpG dinucleotide frequency appears to be an important constraint on codon selection which may also provide reduced susceptibility to APOBEC hotspot mutations. Thus, an apparently vulnerable genome may still adapt some level of resistance to APOBECs by adjusting its CpG usage accordingly, although we only observed this for two vulnerable viral genomes.

We demonstrate that a high GC content and the corresponding observed codon biases are a mechanism by which resistance is conferred (Fig 4 and S6 Fig). Not all hotspot resistance is explained by GC content, and we observe that varying the GC content of random sequences does not change susceptibility to the hAPOBEC3G hotspot CCC. Under our model of susceptibility, which is based upon the percentile of the fraction of nonsynonymous mutations, CCC may be robust to changes in GC content. For example, deamination at a CCC hotspot codon to CCT might be synonymous, whereas if the hotspot were on the opposite strand (and in frame) forming a GGG codon, then deamination to AGG will be nonsynonymous. Since the frequency of both CCC and GGG codons will presumably change linearly with GC content then the fraction of nonsynonymous mutations should remain approximately unchanged for different GC levels. Although this argument applies only when the hotspot is in frame, in practice we find that CCC hotspots are indeed robust to changes in GC content regardless (S3A Fig). Thus, there may be alternating roles for the different APOBEC hotspots, with human APOBECs that prefer NTC to be primed to target low GC content virus sequences and retrotransposons, and human APOBEC3G which may have evolved the CCC hotspot to be robust to the protection a high GC content may confer to other hotspots. Notably, human APOBEC3G is known to restrict HSV-1 in vitro [31], which is a high GC content virus [65]. Due to the differences in susceptibility to the hAPOBEC3G hotspot CCC and the hAPOBEC3B hotspot NTC, there may be an evolutionary advantage in there being multiple APOBEC hotspots since this increases the breadth of viruses (meaning variability in GC content) that the host can optimally restrict. This result is consistent with previous suggestions that the diversification of APOBEC3 genes has arisen due to an arms race against a wide variety of viral pathogens, given that many of the diversified APOBEC3 genes are under positive selection [2, 76, 77].

Our model and definition of susceptibility examines the impact of mutations on both strands, weighing both equally. This model has been sufficient to demonstrate overall trends and differences between a variety of mammalian and non-mammalian genes and viruses. However, in the particular case of retroviruses, APOBEC deamination is biased in that mutations are predominantly G-to-A on the plus strand, as has been observed for HIV and HTLV [78]. Thus, an alternative model that might be considered would examine susceptibility only on one strand. Under this scenario, differences in susceptibility between different retroviruses and among viruses from different clinical isolates might be used as additional evidence of an ongoing arms race between the deaminase and the retrovirus.

Although mutations are likely to be predominantly deleterious, viruses in particular need to maintain variation at the population level, and often within an individual host to escape host immunity. For HIV in particular, there is growing evidence that APOBEC-mediated mutagenesis may actually contribute to the generation of escape variants [27, 64]. Based on the idea that there is an optimal mutation level for viruses to propagate [79], it is conceivable that APOBEC susceptibility may benefit many viruses, particularly if we were to consider individual genes (that contain epitopes, for example) rather than entire genomes as we have done here. Many mammalian genes often showed great differences in susceptibilities to different hotspots as well. In the future, predicting which genes are particularly susceptible may illuminate the overall impact of AID/APOBEC mutagenesis on the mammalian genome as well. Our results suggest that the current AID/APOBEC hotspots are effective in mutating unadapted viruses and retrotransposons, while minimizing collateral damage. However, several of the viruses that we expected would be vulnerable are resistant and vice versa (such as vulnerable HIV, MHV4, and SFV, which are all mammalian viruses). These features may be explained by alternative APOBEC evasion mechanisms, such as the HIV gene *vif*. A similar mechanism was recently proposed to exist for MHV4 [63]. We expect future work, that uses more sophisticated models of sequence evolution and mutation and on more thorough strains of viruses, to elucidate these differences further.

## Methods

### Calculating susceptibility

We describe a model that calculates the susceptibility, U_H_, to motif H, from NNC to NNT, a wildtype coding sequence (denoted as s_wt_), that contains L codons as follows. Given s_wt_, we generate a vector:

$${\text{C}}_{\text{L}}=\;\left({\text{c}}_{1},{\text{c}}_{2},\ldots {\text{c}}_{\text{L}}\right)$$

where c_i_ is the *i*th codon in s_wt_. We generate a set *W*, of N (in practice 1000) random sequences: *W* = (s_1_, s_2_, … s_N_) where each sequence s_j_, in *W* is a sequence generated from s_wt_ by randomizing codon usage as follows. For each sequence s_j_, for each codon c_i_ in C_L_ we replace the codon with another synonymous codon chosen either with uniform probability or a biased probability derived from a GC-bias parameter (see next paragraph). For each sequence s_j_, the *k* occurrences of (real or hypothetical) hotspot motif H are counted. Then for each sequence s_j_ we associate with it a vector H_k_ = (h_1_, h_2_, … h_k_), where h_t_ is the position of the *t*_th_ occurrence of hotspot motif H on sequence s_j_, in both orientations. We define a function f(h_t_) which is 1 if changing the nucleotide at position h_t_ from C to T (or G to A), causes an amino acid change, and is 0 otherwise. Then the nonsynonymous mutation fraction of sequence s_j_ is:

$$\text{M}({\text{s}}_{\text{j}})\;=\;\frac{\sum_{t=1}^{k}f(h_{t})}{k}$$

The susceptibility, U_H_ of wildtype sequence s_wt_ to the hotspot H, is calculated as:

$${\text{U}}_{\text{H}}\;=\;\frac{\sum_{l=1}^{N}\text{R}(l)}{N}$$

Where R(*l*) = 1 if M(S*_l_*) < M(s_wt_), (M(s_wt_) being the nonsynonymous mutation rate of the wild type sequence), and 0 otherwise.

Additionally, we may generate random sequences taking into account GC content, by assigning a weight to each synonymous codon. Given a GC content (fraction) of p, and a set of synonymous codons for a particular amino acid (e.g. GAG and GAA for Glu), a weight is derived for each codon based on the GC content of the codon. Within each codon, we assume that G and C nucleotides are chosen according to GC content with probability p, whereas A and T nucleotides are chosen with probability 1-p. The codon weight is the product of the three corresponding probabilities. Thus, for Glu, the codon GAG would be assigned a weight of p×(1-p)×p = (1-p)p^2^, whereas the codon GAA would be assigned the weight p(1-p)^2^. Codons are then selected with probability equal to a normalized vector of these weights. Note that if p = 0.5, each synonymous codon is selected with equal probability.

### The effect of GC content on susceptibility

As described herein, susceptibility is impacted in many cases by GC content (Fig 4). We evaluated the larger gene sets on a gene-by-gene basis to assess the impact of GC content on susceptibility. These large gene sets include the human and mouse housekeeping gene sets and several virus genomes (S6 Fig). Although not every hotspot we examined demonstrated a strong relationship, the TTC motif had the strongest mean correlation among the 16 NNC motifs we examined, in the human and mouse housekeeping gene sets (Spearman correlation = -0.728 and -0.486 respectively, S6A and S6B Fig). For many of the viral genomes we analyzed, the GC content fell within a very narrow range and/or had very few genes, leading to weak correlations. We therefore grouped the genomes of EBV, HSV-1, Murid Herpesvirus 2, and the Ostreid herpesvirus, as well as many non-herpesviruses with 10 or fewer genes including HIV, Adenoviruses, and others to obtain a wider range of GC content across many genomes. By analyzing all the genes together we observed a trend similar to that for the housekeeping genes, where GC content of the gene correlates with the motif susceptibility (-0.493 for the TTC hotspot, S6C Fig). Because GC content influences susceptibility in some cases, but not others (Fig 4B), we chose in the first instance to keep our analysis neutral and we generated our null model without any constraints on GC, uniformly choosing each synonymous codon at random. We subsequently compared our susceptibility results to the GC content of the corresponding gene set to determine the extent to which GC content determines susceptibility (Fig 4B).

### Controlling for CpG dinucleotide content

To control for CpG dinucleotide frequency we are interested in calculating susceptibility for each NNC motif conditioned on the CpG frequency in the original sequence. It is usually impractical to compute this conditional probability empirically. Controlling for this condition might be achieved by considering only those randomly generated sequences that have, by chance, produced the same number of CpG motifs as the original sequence. However, in practice the fraction of random sequences that exactly match the CpG motif count is often very small, particularly if the CpG content is already skewed, making it unlikely that such exact matches are produced by the random model. This may lead to very small numbers, making an approximation preferable. Thus, we approximated the joint distribution of CpG dinucleotide frequency and susceptibility, assuming the joint distribution is a bivariate normal distribution. The corrected susceptibility is simply the percentile of the approximated Gaussian conditional on the CpG frequency in the analyzed sequence. Correcting for CpG frequency made little difference to our results.

### Data sources

We obtained protein-coding sequences from a variety of biological sources, and broadly speaking our data sources are categorized into three groups: Known or suspected AID/APOBEC targets including viruses and repeat elements; non-targeted viruses; and putative unintentional or collateral targets of AID/APOBEC which include host genes. Known virus targets that we analyzed included HSV-1, AAV-2, HERV (specifically the HCML-ARV retrovirus) and SFV. Since papillomaviruses are highly diverse we selected a well- characterized strain with medical relevance, which was HPV16 due to its link to cervical cancer. We also analyzed EBV, due to its shared B-cell tropism with AID. MHV68, a close relative of the other herpesviruses we studied, is not suspected to be restricted by mAPOBEC3 although multiple human APOBECs were capable of restricting the virus [63], so to test the possibility that mAPOBEC3 hotspots may have impacted murid herpesvirus coding sequence vulnerability or vice versa we included both Murid herpesvirus 2 and MHV68. As controls, we included several viruses unlikely to be restriction targets, due to not being vertebrate or mammalian viruses, which consist of the Ostreid herpesvirus, as well as two plant viruses, the cherry and strawberry viruses (Table 2). These controls were evaluated by substituting other potential controls consistent with the same rationale, i.e. they are not restricted or exposed to AID/APOBEC. We also analyzed a set of potential mutational targets in the mouse genome, namely a data set that is enriched in potentially oncogenic AID off-target genes [56] and sets of housekeeping genes from the human and mouse genomes (see below). Altogether we examined a total of 22 distinct gene sets. We wanted to avoid including an overwhelming number of controls, but at the same time we checked whether those we did include were unusual. Therefore, additional control viruses including plant viruses were obtained and individually verified by including these viruses in the clustering analysis (Fig 3) and determining whether they clustered consistently with the viruses originally used as controls. In each case these clustered with our previous control viruses, demonstrating the validity of the original choices (S7 Fig). Additional controls used to validate our current control set included: Tobacco mosaic virus (NC_001367), Grapevine leafroll associated virus 1 and 3 (NC_016509, NC_004667), Citrus tristeza Virus (NC_001661), Carrot yellow leaf Virus (NC_013007), and Fly C virus (NC_001834). Housekeeping genes for the vertebrate zebrafish, which lacks APOBEC3, and the invertebrate *C*. *elegans* were obtained from a RT-PCR array (http://www.sabiosciences.com/rt_pcr_product/HTML/PAZF-000Z.html) and a study of *C*. *elegans* housekeeping genes respectively [2, 80]. The list of human housekeeping genes were identified in a previous study [81]. In cases where a gene had multiple splice variants, we used the longest sequence available (UCSC Genes track/ knownGenes table via the UCSC genome browser query tool). For the set of mouse housekeeping genes, we used a previously published list which assigned probabilities of being housekeeping to each gene [82]. We used a threshold probability of 0.75 in order to obtain a gene set of comparable size to our human housekeeping gene set. Again, the longest sequence was used in case one gene had several coding sequences. L1 sequences for both mouse and human were downloaded from the UCSC genome browser database under the RepeatMasker table from the *mm10* and *hg19* databases respectively. Sequences were filtered with a minimum of 5500 base pairs, to ensure only full length elements, which are canonically around 6000 bp long. ORFs were extracted using EMBOSS *getorf*, with a minimum length of 1000 nucleotides finding all sequences between start and stop codons. These ORFs were separated based on their family and all ORFs were subsequently separated by family. In total 14 families were identified, including members from the L1PA and L1PB families identified in [52]. For each family, sequences were clustered. Representative sequences were processed to reduce statistical redundancies between repetitive sequences using cd-hit with default parameters [83]. The final L1 set was obtained by merging the representative sequences from each cluster.

### Clustering algorithm

The data being clustered was a matrix of the average susceptibility scores, which range from 0 to 1, of all 16 NNC motifs in the columns, and gene sets (e.g. L1, Housekeeping genes, etc.) in the rows. Thus, both rows and columns were clustered using standard hierarchical agglomerative clustering and average linkage, using the Euclidean distance metric as the distances between each row and column. These clusters were computed and displayed using the heatmap.2 function in the R library gplots. We further validated these clusters using a k-means approach, showing that when we tested across a range of values for k, 2 clusters represents an optimal separation of the gene sets, indicated by the largest decline in intra-cluster sum of squares (S8A Fig). As an alternative approach, we used Principal Component Analysis (PCA). Here, one principal component accounts for a large amount of the overall trend in motif susceptibility (S8B Fig), and the clusters of gene sets are consistent for the different methods and separate primarily along the first principal component (S8C Fig).

Codon bias is likely correlated with the more general measure of trinucleotide (NNN) frequency. We confirmed this by calculating the trinucleotide frequency usage of all of our gene sets and clustering them similarly (S9 Fig). The occurrence of the trinucleotide AAA was skewed for the L1 elements for human and mouse, so it was excluded as an outlier. We observed the same clusters as before in Fig 3, except that Hepatitis B virus, and HERV, which under susceptibility are grouped with the vulnerable clusters that include L1 elements among others, now appears in the resistant cluster that includes housekeeping genes and mammalian viruses.

### Statistical sampling of hotspot ranks

To assess statistically whether the rankings of certain groups of the 16 possible NNC (for example, the 4 human APOBEC3B NTC hotspots of ATC, CTC, GTC, and TTC) motifs were significantly high or low, we performed a pairwise comparison between each of the 10 resistant and the 12 vulnerable genomes of Fig 3. For each comparison, we ranked the motifs by the difference in average susceptibilities between the resistant and the vulnerable gene set (resistant-vulnerable), noting the ranks of the 4 motifs in each comparison. We assessed significance by sampling one resistant and one corresponding vulnerable gene set chosen at random, until one of each resistant gene set was sampled, and ensuring no gene set was sampled more than once. In each comparison we noted the ranks of the 4 motifs, summed them and compare the sum to a random null model selection of ranks, taken 4 at a time uniformly from the numbers 1 through 16 without replacement. For example, if examining the statistical significance of the motif NTC, the ranks of the hotspots ATC, CTC, GTC, and TTC, for a particular resistant vs vulnerable gene set, were 1, 3, 5, and 9, these would be compared to a random selection of numbers 1–16 which could be 3, 8, 9, 15. The sums of these ranks would be 18 and 35 respectively. We then summed the ranks of the 4 hotspots across all our comparisons and compared this to the sum of null model ranks. Repeated 10000 times, our bootstrapped P-value was the fraction of times the sum of observed ranks found to be greater than the sum of the random ranks. If this fraction (equivalent to a P-value) were greater than 0.05, this demonstrated that the observed ranks were not significant.

## Supporting information

S1 Fig Side-by-side comparison of susceptibilities accounting for an alternate measure correcting GC bias. A) Without correcting for GC content (same as Fig 3) and B) with correcting for GC content based on the GC content of each genome’s coding sequence (right), but with the ordering of the motifs and gene families kept intact. Although the contrasts in susceptibilities are not as stark with GC accounted for, they still are in agreement with the non-corrected case, suggesting an additional role of gene susceptibility beyond adjusting GC content.
(PDF)

S2 Fig Measuring NNC hotspot susceptibility under alternate mutation models and controls. A) Underrepresentation of non-synonymous mutation count (rather than rate). In this alternate model, the gene sets form two distinct clusters that are the same as the resistant and vulnerable clusters observed under our regular model (Fig 3). However, the resistant cluster in bottom shows higher susceptibilities (in green) to GC rich hotspots such as CCC and GGC and the vulnerable cluster on top shows lower susceptibilities (in red) to these same hotspots. B: Clustering of susceptibility (minimizing nonsynonymous mutation rate fraction) for hypothesized NNA to NNG, C: NNT to NNC hotspots, D: NNG to NNA, and E: NNC to NNG hotspots. NNA to NNG and NNT to NNC trends are reversed from NNC to NNT, but not NNG to NNA suggesting that C or G to non-C/G mutations are optimal for host genomes.
(PDF)

S3 Fig Effects of GC content, codon bias, and selection pressure on susceptibilities. A) Susceptibility of completely random sequences of various GC weights suggest a higher GC content confers resistance to most observed APOBEC hotspots, except CCC. B) Existing coding sequences of OsHV (vulnerable) and EBV (resistant) genomes are influenced by codons weighted by various GC count. C) By generating synthetic sequences of existing genomes using a specific codon bias and calculating susceptibility, we discovered that Codon bias of EBV and OsHV confers appropriate susceptibilities to many hotspots. The codon bias of L1 elements similarly confers higher susceptibility than that of human housekeeping genes. D) Weighted mutations favored at the first two positions (when the modeled selective pressure parameter, which is correlated to dN/dS, is high) do not alter sequence susceptibility (taken as the average of all 16 NNC motif susceptibilities) strongly.
(PDF)

S4 Fig Simulations of mutations at hotspots suggest how susceptibilities can change over time. A) Mutations at AID hotspots using a full model of AID somatic hypermutation (SHM) change susceptibility to virus sequences to be more resistant. B) Mutations at AID hotspots using a modified model of AID hypermutation only mutating C to T and G to A change susceptibilities of viruses to be weaker, suggesting hypermutation at hotspots alone initially weaken a sequence. C) Mutations at APOBEC3B hotspots (TC) and D) APOBEC3G hotspots (CCC) using a simplified model of mutating a fraction of the observed hotspots at random from C to T showing changes in the native gene susceptibilities over time.
(PDF)

S5 Fig Underrepresentation of non-synonymous mutation fraction, corrected for the dinucleotide CpG, an important marker. As with our regular model (Fig 3), our gene sets cluster into broadly vulnerable (top cluster) and resistant (bottom cluster). However under this model of susceptibility, two vulnerable gene sets are now reclassified as resistant.
(PDF)

S6 Fig The GC content of a virus gene is predictive of its non-synonymous mutation rate to the hotspot TTC, which defines susceptibility. Correlation of individual genes of a gene’s GC content (X axis) and its observed non-synonymous mutation rate for the hotspot TTC (Y axis), for A) Human housekeeping genes, B) Mouse housekeeping genes, and C) many viruses. A differing GC content influences the non-synonymous mutation rate for certain hotspots.
(PDF)

S7 Fig Verification of the observed APOBEC-vulnerable cluster in our standard analysis (Fig 3). Additional invertebrate and plant viruses, with hosts that do not have a native APOBEC, were added to determine the statistical validity of the initial vulnerable cluster (top cluster). In all cases the additional gene sets we added to determine the strength of the clusters were grouped in with other known vulnerable gene sets.
(PDF)

S8 Fig Mathematical validation of clustering gene sets by hotspot susceptibility. A) A k-means approach to clustering gene sets suggest that two clusters is ideal. B) One principal component accounts for a majority of the trend of hotspot susceptibility. C) Clusters along this principal component confirm same patterns of vulnerable (blue) and resistant (red) gene sets.
(PDF)

S9 Fig Clustering of resistant and vulnerable gene sets based on the 63 trinucleotide frequencies (excluding AAA, an outlier) in each gene set. The clusters are similar but not identical to that in our main finding, suggesting a correlation between codon bias and APOBEC resistance.
(PDF)

S1 Table Fraction of significant (using unweighted z-score) “resistant” genes with statistical significance. (XLSX)

S2 Table Fraction of significant (using unweighted z-score) "vulnerable" gene sets with statistical significance. (XLSX)

S3 Table Statistically significant vulnerable and resistant gene sets with a GC corrected susceptibility measure calculated. (XLS)

S4 Table Comparison of human housekeeping to human L1 susceptibilities to different NNC hotspots. (XLSX)

S5 Table Average rank of motifs in comparisons between different gene sets. (XLSX)

S6 Table Comparison of EBV gene set to OSHV gene set susceptibilities to different NNC motifs. (XLSX)
