# Supplementary material for: The preferred nucleotide contexts of the AID/APOBEC cytidine deaminases have differential effects when mutating retrotransposon and virus sequences compared to host genes
Source: PLoS Comput Biol. 2017 Mar 31;13(3):e1005471. doi: 10.1371/journal.pcbi.1005471 (PMC5391955; doi:10.1371/journal.pcbi.1005471)
Supplement: S1 Fig — A) Without correcting for GC content (same as Fig 3) and B) with correcting for GC content based on the GC content of each genome’s coding sequence (right), but with the ordering of the motifs and gene families kept intact. Although the contrasts in susceptibilities are not as stark with GC accounted for, they still are in agreement with the non-corrected case, suggesting an additional role of gene susceptibility beyond adjusting GC content. (PDF) [file pcbi.1005471.s001.pdf]

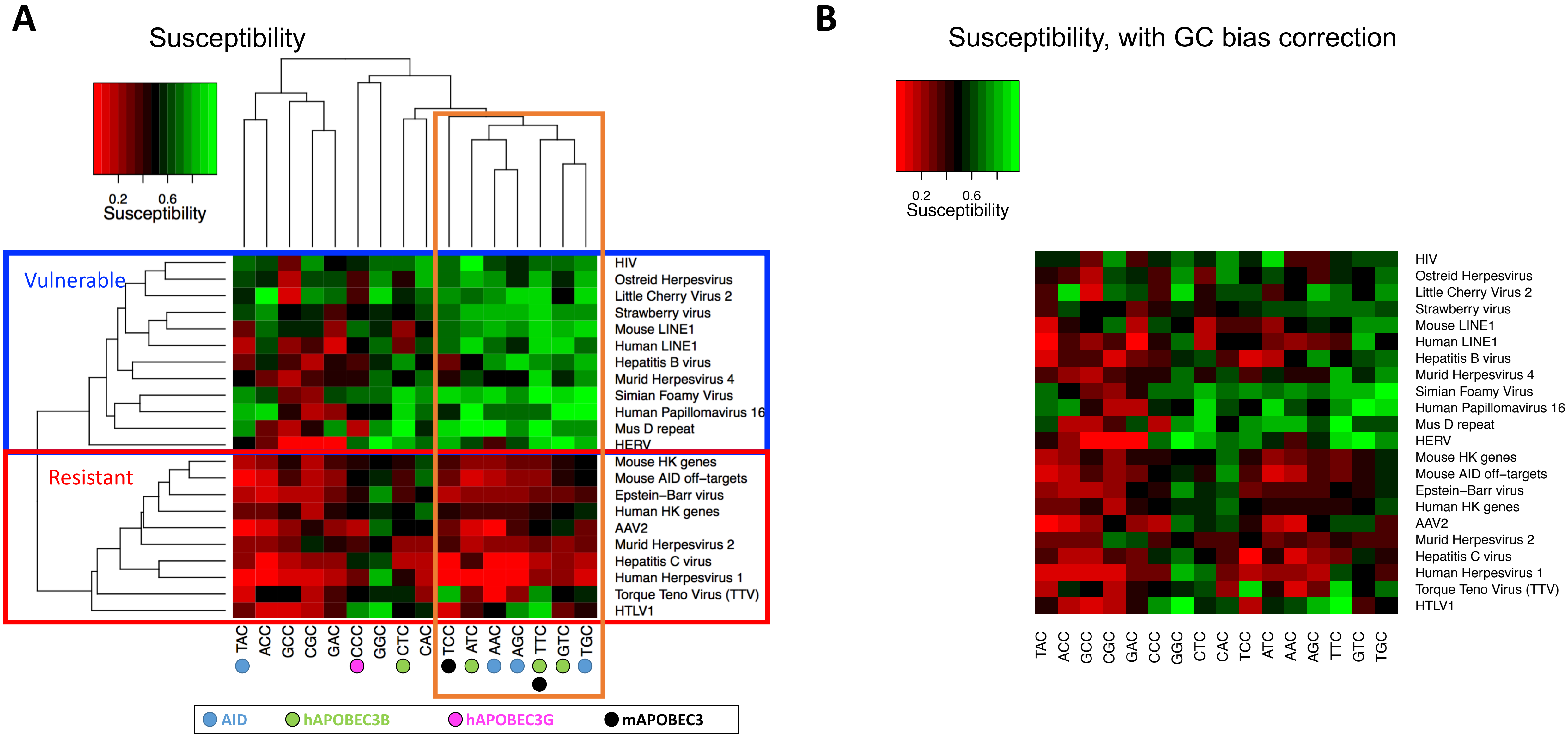

S1 Fig- **Side-by-side comparison of susceptibilities accounting for an alternate measure correcting GC bias.** A) Without correcting for GC content (same as Fig 3) and B) with correcting for GC content based on the GC content of each genome's coding sequence (right), but with the ordering of the motifs and gene families kept intact. Although the contrasts in susceptibilities are not as stark with GC accounted for, they still are in agreement with the non-corrected case, suggesting an additional role of gene susceptibility beyond adjusting GC content.
