# Supplementary material for: The preferred nucleotide contexts of the AID/APOBEC cytidine deaminases have differential effects when mutating retrotransposon and virus sequences compared to host genes
Source: PLoS Comput Biol. 2017 Mar 31;13(3):e1005471. doi: 10.1371/journal.pcbi.1005471 (PMC5391955; doi:10.1371/journal.pcbi.1005471)
Supplement: S3 Fig — A) Susceptibility of completely random sequences of various GC weights suggest a higher GC content confers resistance to most observed APOBEC hotspots, except CCC. B) Existing coding sequences of OsHV (vulnerable) and EBV (resistant) genomes are influenced by codons weighted by various GC count. C) By generating synthetic sequences of existing genomes using a specific codon bias and calculating susceptibility, we discovered that Codon bias of EBV and OsHV confers appropriate susceptibilities to many hotspots. The codon bias of L1 elements similarly confers higher susceptibility than that of human housekeeping genes. D) Weighted mutations favored at the first two positions (when the modeled selective pressure parameter, which is correlated to dN/dS, is high) do not alter sequence susceptibility (taken as the average of all 16 NNC motif susceptibilities) strongly. (PDF) [file pcbi.1005471.s003.pdf]

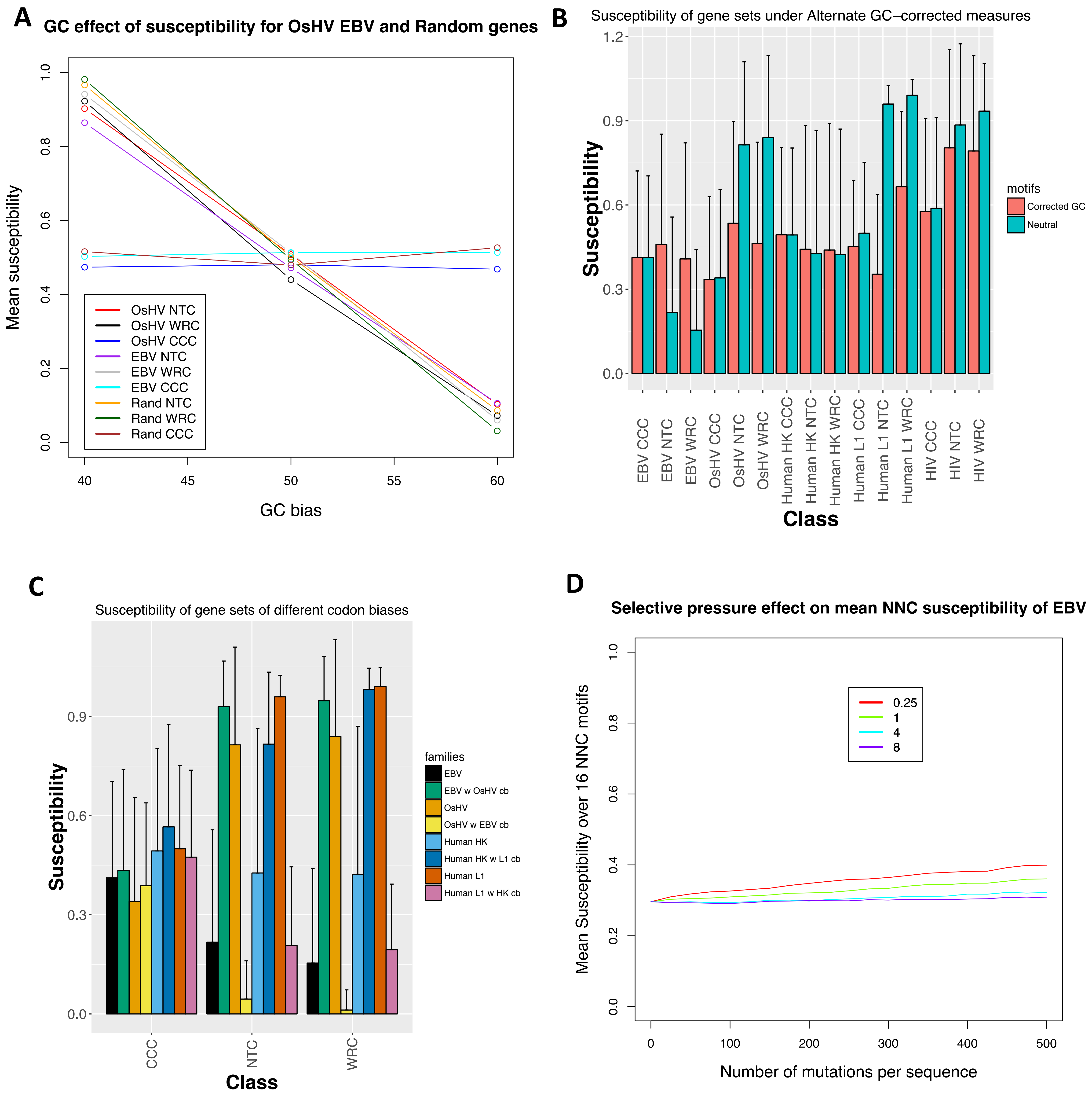

S3 Fig- **Effects of GC content, codon bias, and selection pressure on susceptibilities.** A) Susceptibility of completely random sequences of various GC weights suggest a higher GC content confers resistance to most observed APOBEC hotspots, except CC<u>C</u>. B) Existing coding sequences of OsHV (vulnerable) and EBV (resistant) genomes are influenced by codons weighted by various GC count. C) By generating synthetic sequences of existing genomes using a specific codon bias and calculating susceptibility, we discovered that Codon bias of EBV and OsHV confers appropriate susceptibilities to many hotspots. The codon bias of L1 elements similarly confers higher susceptibility than that of human housekeeping genes. D) Weighted mutations favored at the first two positions (when the modeled selective pressure parameter, which is correlated to dN/dS, is high) do not alter sequence susceptibility (taken as the average of all 16 NNC motif susceptibilities) strongly.
