# Supplementary material for: The preferred nucleotide contexts of the AID/APOBEC cytidine deaminases have differential effects when mutating retrotransposon and virus sequences compared to host genes
Source: PLoS Comput Biol. 2017 Mar 31;13(3):e1005471. doi: 10.1371/journal.pcbi.1005471 (PMC5391955; doi:10.1371/journal.pcbi.1005471)
Supplement: S4 Fig — A) Mutations at AID hotspots using a full model of AID somatic hypermutation (SHM) change susceptibility to virus sequences to be more resistant. B) Mutations at AID hotspots using a modified model of AID hypermutation only mutating C to T and G to A change susceptibilities of viruses to be weaker, suggesting hypermutation at hotspots alone initially weaken a sequence. C) Mutations at APOBEC3B hotspots (TC) and D) APOBEC3G hotspots (CCC) using a simplified model of mutating a fraction of the observed hotspots at random from C to T showing changes in the native gene susceptibilities over time. (PDF) [file pcbi.1005471.s004.pdf]

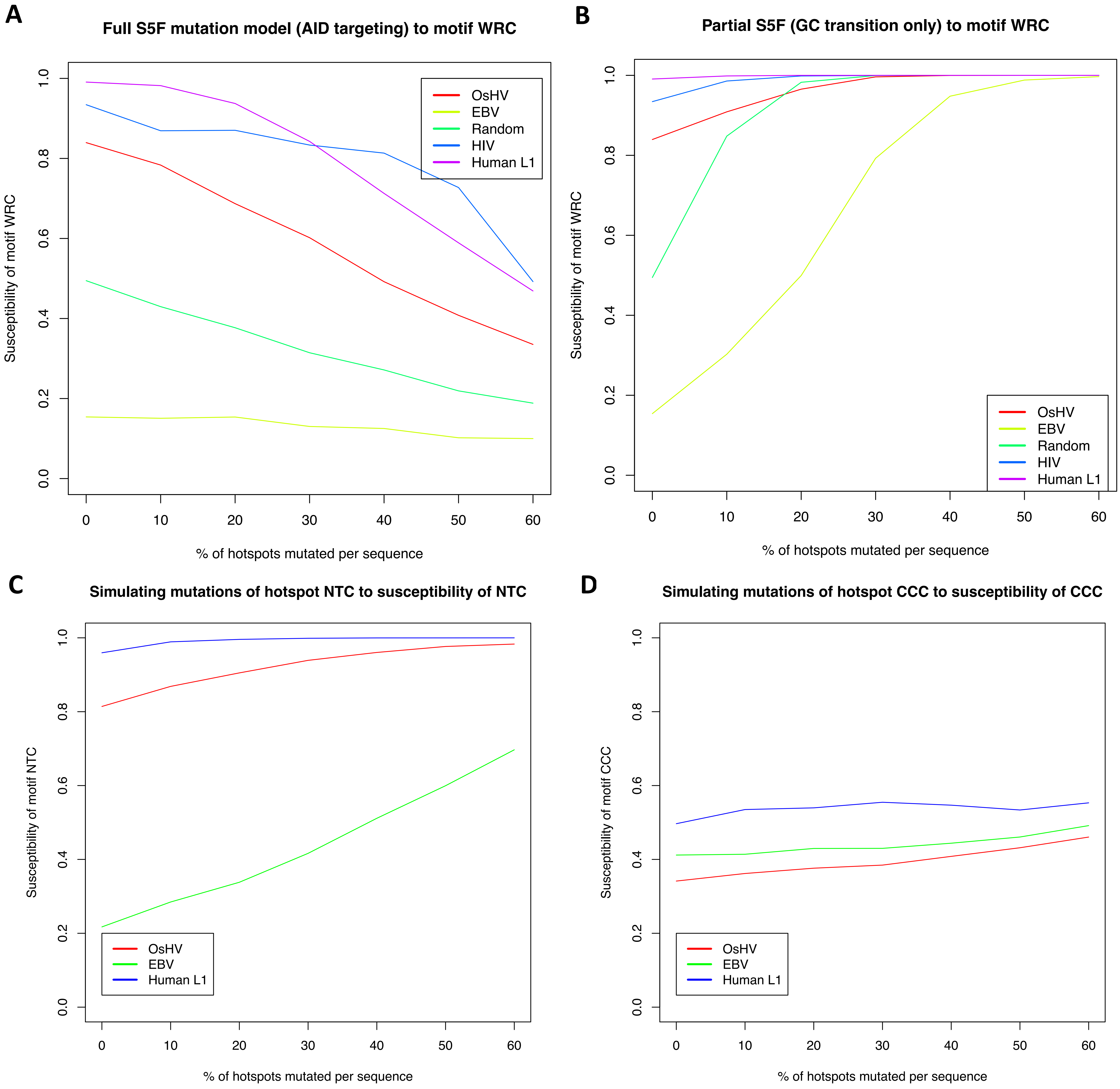

S4 Fig- **Simulations of mutations at hotspots suggest how susceptibilities can change over time.** A) Mutations at AID hotspots using a full model of AID somatic hypermutation (SHM) change susceptibility to virus sequences to be more resistant. B) Mutations at AID hotspots using a modified model of AID hypermutation only mutating C to T and G to A change susceptibilities of viruses to be weaker, suggesting hypermutation at hotspots alone initially weaken a sequence. C) Mutations at APOBEC3B hotspots (T<u>C</u>) and D) APOBEC3G hotspots (CC<u>C</u>) using a simplified model of mutating a fraction of the observed hotspots at random from C to T showing changes in the native gene susceptibilities over time.
