# Supplementary material for: The preferred nucleotide contexts of the AID/APOBEC cytidine deaminases have differential effects when mutating retrotransposon and virus sequences compared to host genes
Source: PLoS Comput Biol. 2017 Mar 31;13(3):e1005471. doi: 10.1371/journal.pcbi.1005471 (PMC5391955; doi:10.1371/journal.pcbi.1005471)
Supplement: S5 Fig — As with our regular model (Fig 3), our gene sets cluster into broadly vulnerable (top cluster) and resistant (bottom cluster). However under this model of susceptibility, two vulnerable gene sets are now reclassified as resistant. (PDF) [file pcbi.1005471.s005.pdf]

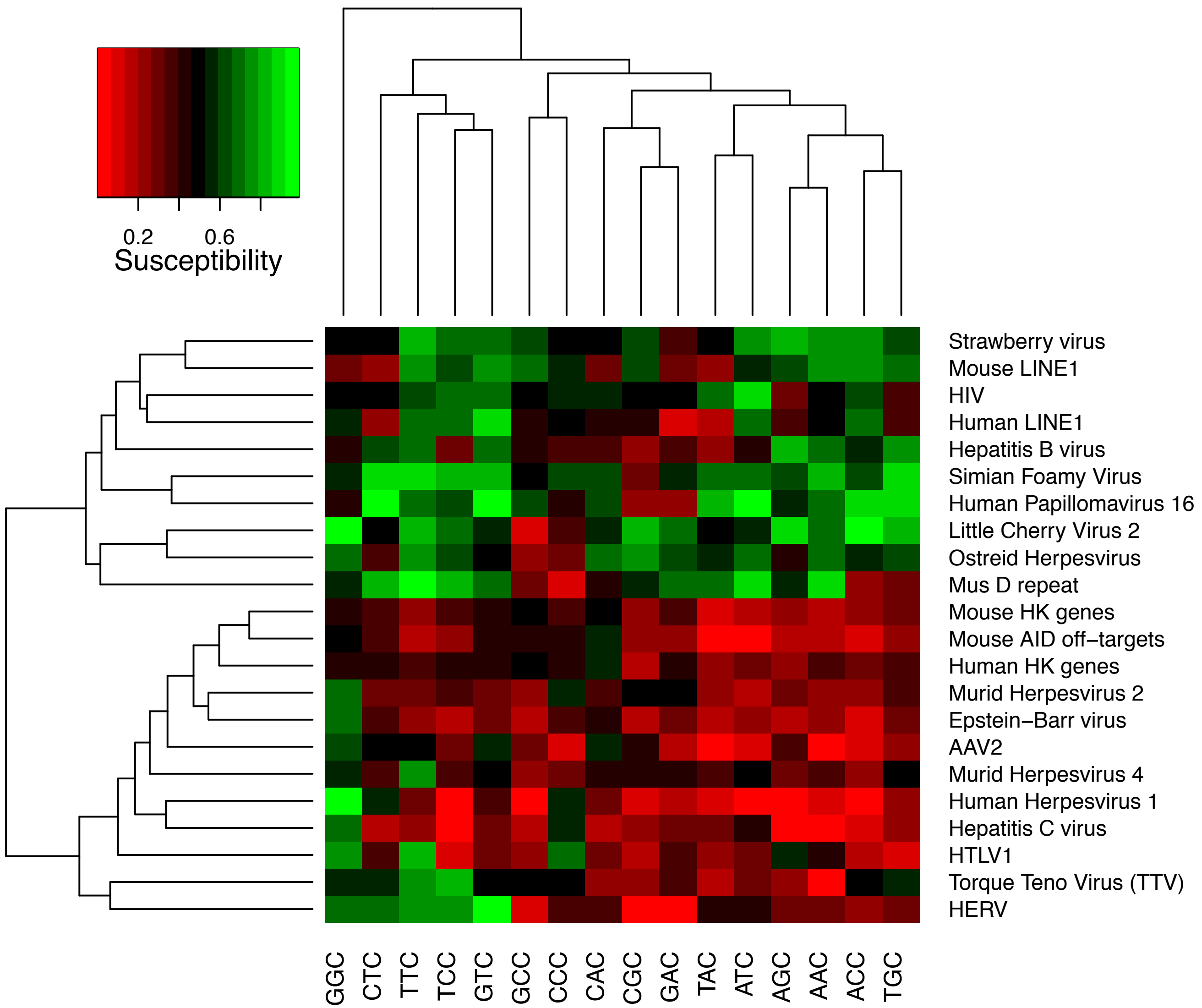

S5 Fig – **Underrepresentation of non-synonymous mutation fraction, corrected for the dinucleotide CpG, an important marker.** As with our regular model (Fig. 3), our gene sets cluster into broadly vulnerable (top cluster) and resistant (bottom cluster). However under this model of susceptibility, two vulnerable gene sets are now reclassified as resistant.
