# Supplementary material for: The preferred nucleotide contexts of the AID/APOBEC cytidine deaminases have differential effects when mutating retrotransposon and virus sequences compared to host genes
Source: PLoS Comput Biol. 2017 Mar 31;13(3):e1005471. doi: 10.1371/journal.pcbi.1005471 (PMC5391955; doi:10.1371/journal.pcbi.1005471)
Supplement: S6 Fig — Correlation of individual genes of a gene’s GC content (X axis) and its observed non-synonymous mutation rate for the hotspot TTC (Y axis), for A) Human housekeeping genes, B) Mouse housekeeping genes, and C) many viruses. A differing GC content influences the non-synonymous mutation rate for certain hotspots. (PDF) [file pcbi.1005471.s006.pdf]

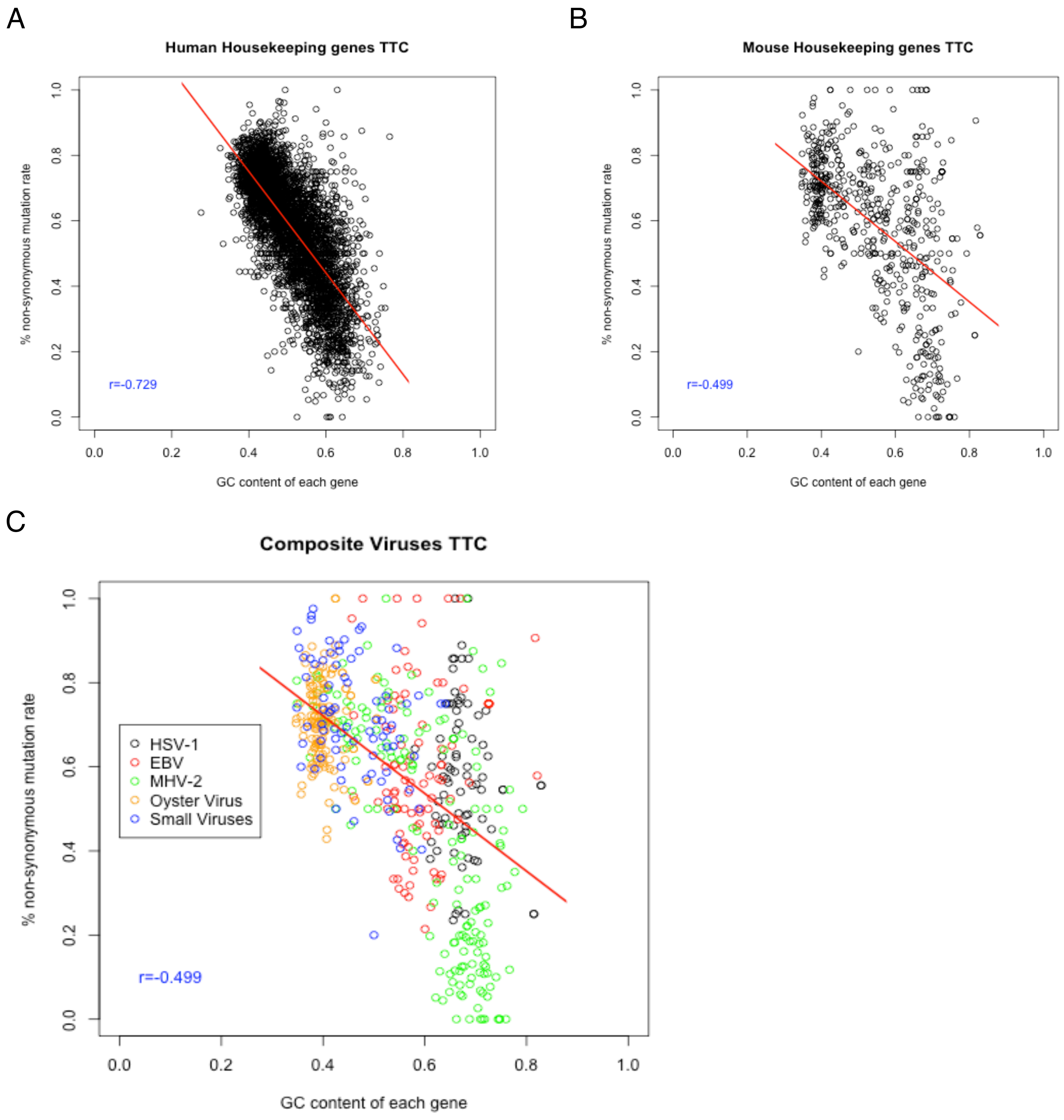

S6 Fig– **The GC content of a virus gene is predictive of its non-synonymous mutation rate to the hotspot TT<u>C</u>, which defines susceptibility.** Correlation of individual genes of a gene's GC content (X axis) and its observed non-synonymous mutation rate for the hotspot TT<u>C</u> (Y axis), for A) Human housekeeping genes, B) Mouse housekeeping genes, and C) many viruses. A differing GC content influences the non-synonymous mutation rate for certain hotspots.
