# Supplementary material for: The preferred nucleotide contexts of the AID/APOBEC cytidine deaminases have differential effects when mutating retrotransposon and virus sequences compared to host genes
Source: PLoS Comput Biol. 2017 Mar 31;13(3):e1005471. doi: 10.1371/journal.pcbi.1005471 (PMC5391955; doi:10.1371/journal.pcbi.1005471)
Supplement: S7 Fig — Additional invertebrate and plant viruses, with hosts that do not have a native APOBEC, were added to determine the statistical validity of the initial vulnerable cluster (top cluster). In all cases the additional gene sets we added to determine the strength of the clusters were grouped in with other known vulnerable gene sets. (PDF) [file pcbi.1005471.s007.pdf]

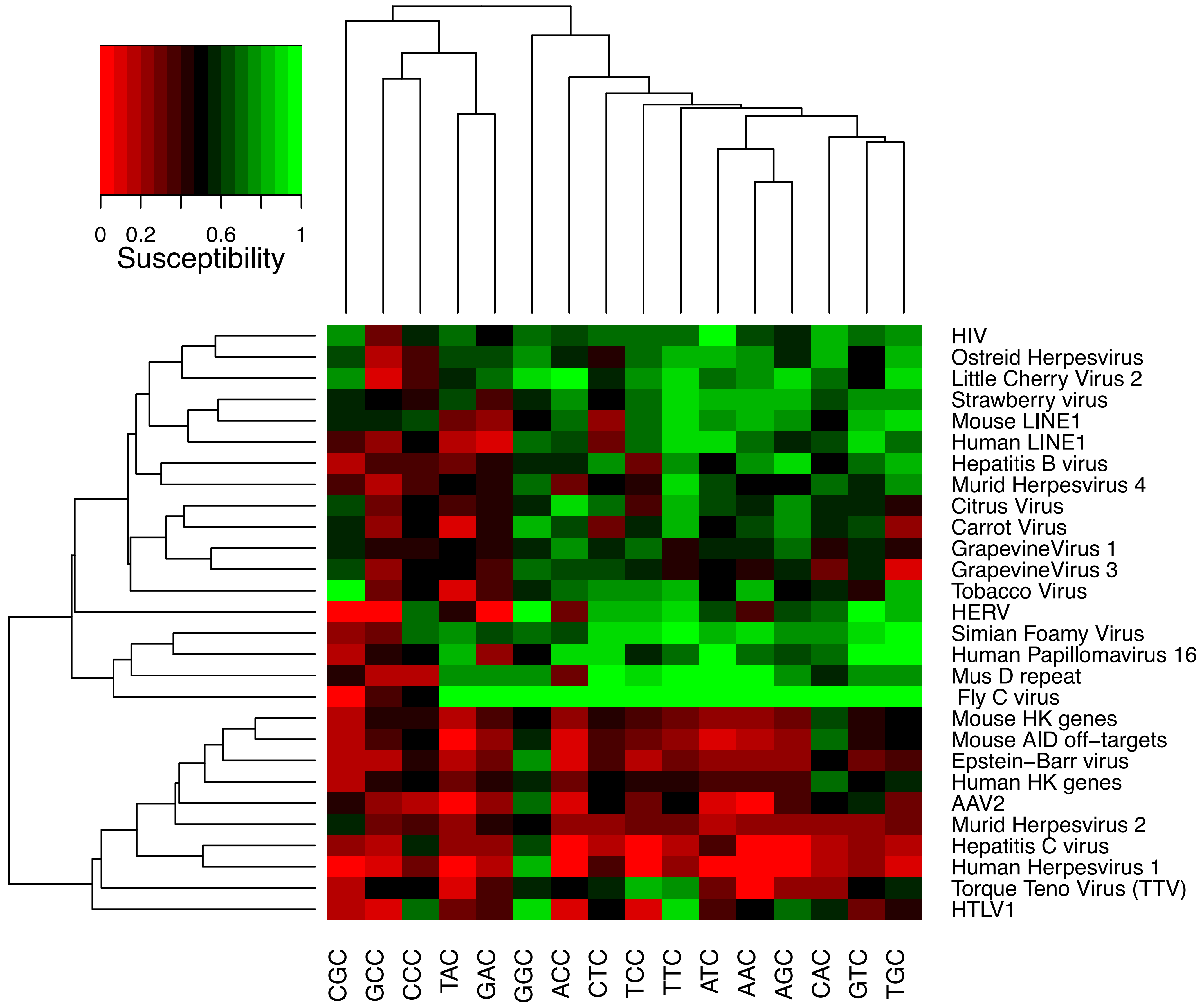

S7 Fig– **Verification of the observed APOBEC-vulnerable cluster in our standard analysis** (Fig 3). Additional invertebrate and plant viruses, with hosts that do not have a native APOBEC, were added to determine the statistical validity of the initial vulnerable cluster (top cluster). In all cases the additional gene sets we added to determine the strength of the clusters were grouped in with other known vulnerable gene sets.
