# Supplementary material for: The preferred nucleotide contexts of the AID/APOBEC cytidine deaminases have differential effects when mutating retrotransposon and virus sequences compared to host genes
Source: PLoS Comput Biol. 2017 Mar 31;13(3):e1005471. doi: 10.1371/journal.pcbi.1005471 (PMC5391955; doi:10.1371/journal.pcbi.1005471)
Supplement: S8 Fig — A) A k-means approach to clustering gene sets suggest that two clusters is ideal. B) One principal component accounts for a majority of the trend of hotspot susceptibility. C) Clusters along this principal component confirm same patterns of vulnerable (blue) and resistant (red) gene sets. (PDF) [file pcbi.1005471.s008.pdf]

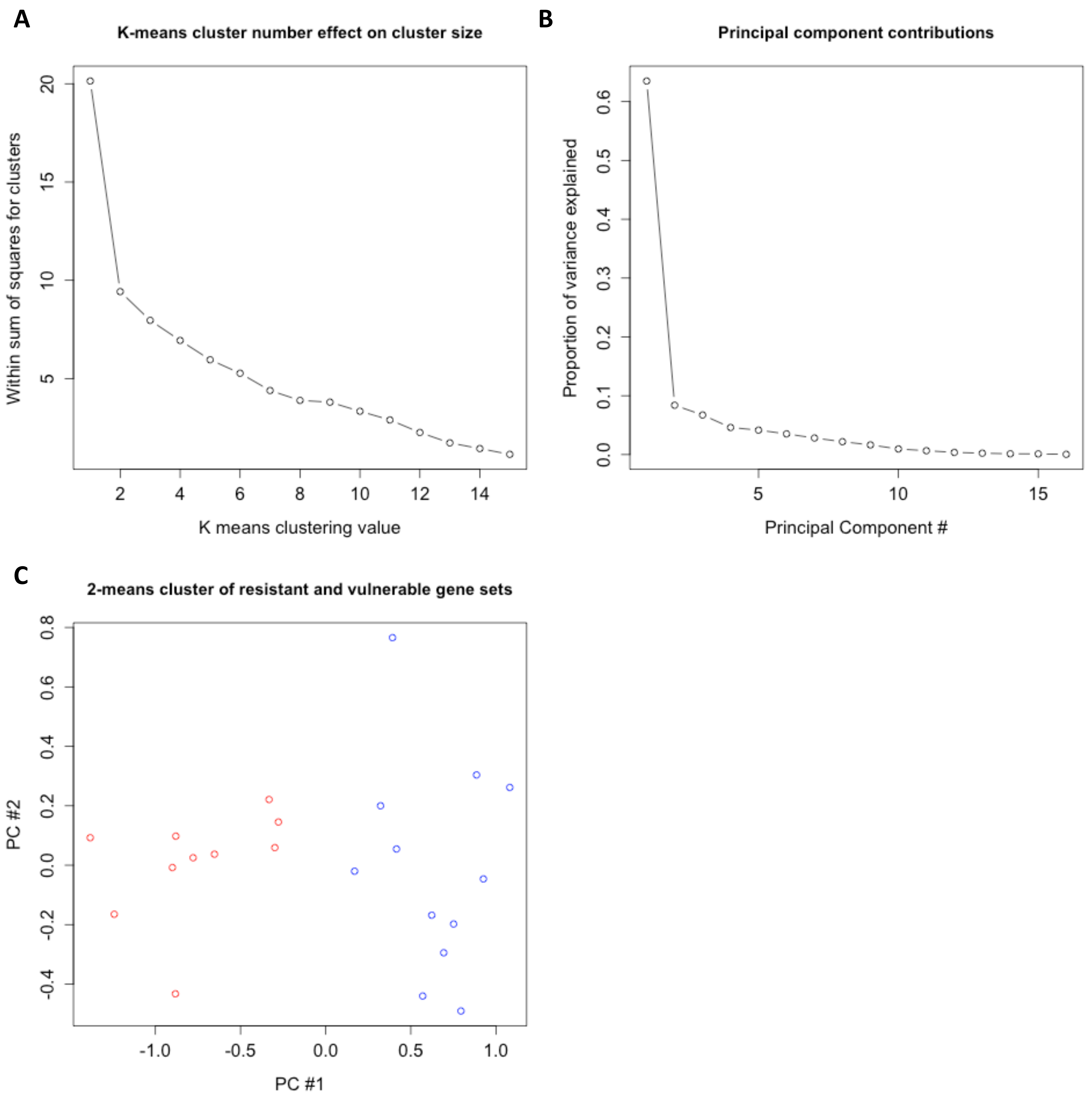

S8 Fig– **Mathematical validation of clustering gene sets by hotspot susceptibility.** A) A k-means approach to clustering gene sets suggest that two clusters is ideal. B) One principal component accounts for a majority of the trend of hotspot susceptibility. C) Clusters along this principal component confirm same patterns of vulnerable (blue) and resistant (red) gene sets.
