# Supplementary material for: The preferred nucleotide contexts of the AID/APOBEC cytidine deaminases have differential effects when mutating retrotransposon and virus sequences compared to host genes
Source: PLoS Comput Biol. 2017 Mar 31;13(3):e1005471. doi: 10.1371/journal.pcbi.1005471 (PMC5391955; doi:10.1371/journal.pcbi.1005471)
Supplement: S2 Fig — A) Underrepresentation of non-synonymous mutation count (rather than rate). In this alternate model, the gene sets form two distinct clusters that are the same as the resistant and vulnerable clusters observed under our regular model (Fig 3). However, the resistant cluster in bottom shows higher susceptibilities (in green) to GC rich hotspots such as CCC and GGC and the vulnerable cluster on top shows lower susceptibilities (in red) to these same hotspots. B: Clustering of susceptibility (minimizing nonsynonymous mutation rate fraction) for hypothesized NNA to NNG, C: NNT to NNC hotspots, D: NNG to NNA, and E: NNC to NNG hotspots. NNA to NNG and NNT to NNC trends are reversed from NNC to NNT, but not NNG to NNA suggesting that C or G to non-C/G mutations are optimal for host genomes. (PDF) [file pcbi.1005471.s002.pdf]

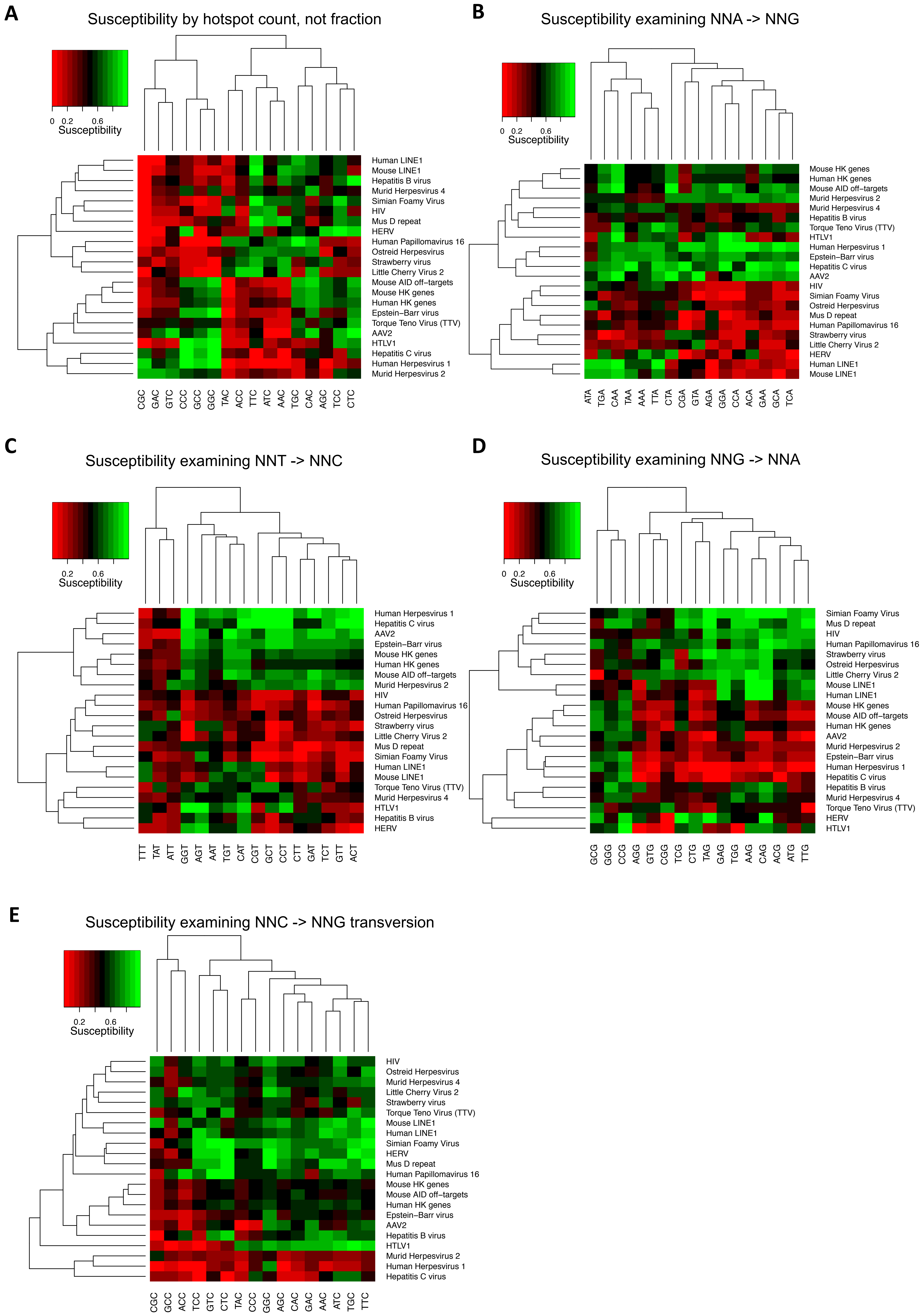

S2 Fig– **Measuring NNC hotspot susceptibility under alternate mutation models and controls.** A) Underrepresentation of non-synonymous mutation count (rather than rate). In this alternate model, the gene sets form two distinct clusters that are the same as the resistant and vulnerable clusters observed under our regular model (Fig. 3). However, the resistant cluster in bottom shows higher susceptibilities (in green) to GC rich hotspots such as CCC and GGC and the vulnerable cluster on top shows lower susceptibilities (in red) to these same hotspots. B: Clustering of susceptibility (minimizing nonsynonymous mutation rate fraction) for hypothesized NNA to NNG, C: NNT to NNC hotspots, D: NNG to NNA, and E: NNC to NNG hotspots. NNA to NNG and NNT to NNC trends are reversed from NNC to NNT, but not NNG to NNA suggesting that C or G to non-C/G mutations are optimal for host genomes.
