# Supplementary material for: The preferred nucleotide contexts of the AID/APOBEC cytidine deaminases have differential effects when mutating retrotransposon and virus sequences compared to host genes
Source: PLoS Comput Biol. 2017 Mar 31;13(3):e1005471. doi: 10.1371/journal.pcbi.1005471 (PMC5391955; doi:10.1371/journal.pcbi.1005471)
Supplement: S9 Fig — The clusters are similar but not identical to that in our main finding, suggesting a correlation between codon bias and APOBEC resistance. (PDF) [file pcbi.1005471.s009.pdf]

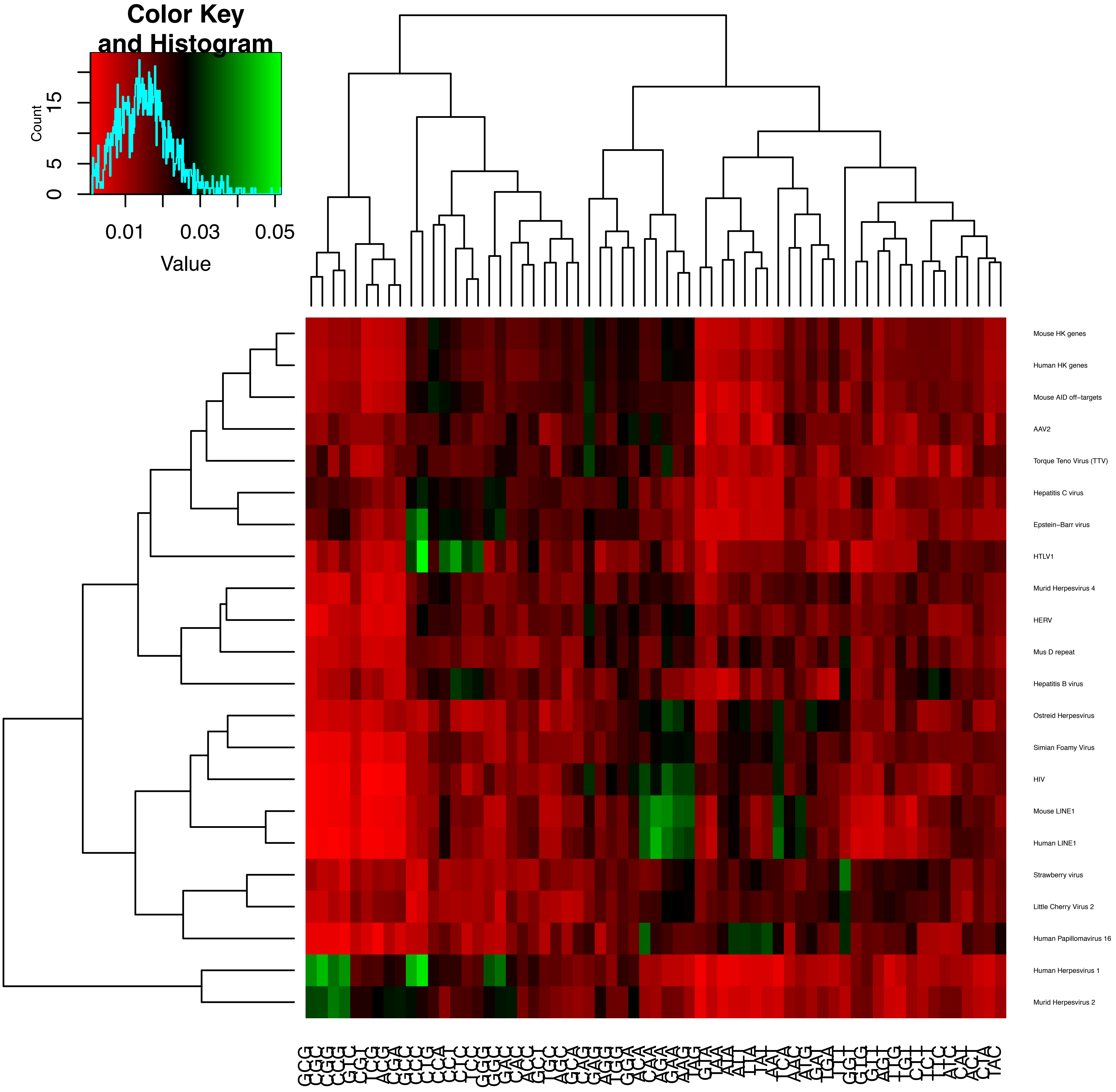

S9 Fig - clustering of resistant and vulnerable gene sets based on the 63 trinucleotide frequencies (excluding AAA, an outlier) in each gene set. The clusters are similar but not identical to that in our main finding, suggesting a correlation between codon bias and APOBEC resistance.
